# Bioactive Phytochemicals of *Citrus reticulata* Seeds—An Example of Waste Product Rich in Healthy Skin Promoting Agents

**DOI:** 10.3390/antiox11050984

**Published:** 2022-05-18

**Authors:** Tarfah Al-Warhi, Abeer H. Elmaidomy, Samy Selim, Mohammad M. Al-Sanea, Mha Albqmi, Ehab M. Mostafa, Sabouni Ibrahim, Mohammed M. Ghoneim, Ahmed M. Sayed, Usama Ramadan Abdelmohsen

**Affiliations:** 1 Department of Chemistry, College of Science, Princess Nourah bint Abdulrahman University, P.O. Box 84428, Riyadh 11671, Saudi Arabia; tialwarhi@pnu.edu.sa; 2 Department of Pharmacognosy, Faculty of Pharmacy, Beni-Suef University, Beni-Suef 62511, Egypt; abeer011150@pharm.bsu.edu.eg; 3 Department of Clinical Laboratory Sciences, College of Applied Medical Sciences, Jouf University, Sakaka 72341, Saudi Arabia; sabdulsalam@ju.edu.sa; 4 Department of Pharmaceutical Chemistry, College of Pharmacy, Jouf University, Sakaka 72341, Saudi Arabia; 5 Olive Research Center, Jouf University, Sakaka 72341, Saudi Arabia; maalbgmi@ju.edu.sa (M.A.); isabouni@ju.edu.sa (S.I.); 6 Pharmacognosy Department, College of Pharmacy, Jouf University, Sakaka 72341, Saudi Arabia; emmoustafa@ju.edu.sa; 7 Pharmacognosy and Medicinal Plants Department, Faculty of Pharmacy (Boys), Al-Azhar University, Cairo 11884, Egypt; mghoneim@mcst.edu.sa; 8 Department of Pharmacy Practice, College of Pharmacy, Al Maarefa University, Ad Diriyah 13713, Saudi Arabia; 9 Department of Pharmacognosy, Faculty of Pharmacy, Nahda University, Beni-Suef 62513, Egypt; ahmed.mohamed.sayed@nub.edu.eg; 10 Department of Pharmacognosy, Faculty of Pharmacy, Minia University, Minia 61519, Egypt; 11 Department of Pharmacognosy, Faculty of Pharmacy, Deraya University, 7 Universities Zone, New Minia 61111, Egypt

**Keywords:** orange, *Citrus*, hyaluronidase, xanthine oxidase, tyrosinase, docking

## Abstract

Phytochemical investigation of Egyptian mandarin orange (*Citrus reticulata* Blanco, F. Rutaceae) seeds afforded thirteen known compounds, **1**–**13**. The structures of isolated compounds were assigned using 1D and 2D NMR and HRESIMS analyses. To characterize the pharmacological activity of these compounds, several integrated virtual screening-based and molecular dynamics simulation-based experiments were applied. As a result, compounds **2**, **3** and **5** were putatively identified as hyaluronidase, xanthine oxidase and tyrosinase inhibitors. The subsequent in vitro testing was done to validate the in silico-based experiments to highlight the potential of these flavonoids as promising hyaluronidase, xanthine oxidase and tyrosinase inhibitors with IC_50_ values ranging from 6.39 ± 0.36 to 73.7 ± 2.33 µM. The present study shed light on the potential of Egyptian mandarin orange’s waste product (i.e., its seeds) as a skin health-promoting natural agent. Additionally, it revealed the applicability of integrated inverse docking-based virtual screening and MDS-based experiments in efficiently predicting the biological potential of natural products.

## 1. Introduction

Mandarins, *C. reticulata*, are some of the most remarkable citrus crops for fresh utilization [1]. They are frequently introduced as ‘tangerines;’ however, ‘mandarin’ is the more popular name. Several hybrids and varieties for mandarin species are distributed in nature [1]. The most famous, sophisticated species consist of *C. unshiu* Marcovitch, *C. nobilis* Loureiro, *C. deliciosa* Tenore and *C. reticulata* Blanco [1,2]. Mandarins are among the principal *Citrus* fruits harvested in many countries. Although the crops are mainly employed for dessert, they possess substantial commercial importance due to their essential oils [3]. *Citrus* flavors are applied in beverage, confectionary, cookies and desserts [4]. Also, *C. reticulata* peel is used for flavorings of liquor [4].

Inverse docking is a type of virtual screening method that can be manipulated to anticipate the potential molecular targets for a given compound structure. Previously, we have utilized this efficient virtual screening method to determine the best protein targets to several isolated natural products [5]. However, the docking scores that resulted from this preliminary screening step only provide some information about the structural complementarity between the protein targets and the query compound structure [6]. Accordingly, molecular dynamics simulation (MDS) experiments, including absolute binding free energy (Δ*G*_binding_) provide more rigorous and trusted results that can be selected for in vitro testing. Herein, we subjected *C. reticulata* seeds to a stepwise chromatographic isolation to get some idea about the major phytochemical in this waste product. Subsequently, we investigated whether those phytochemicals have certain pharmacological effects by subjecting them to a stepwise in silico-based analysis that was initiated by a comprehensive inverse docking and ended by several MDS experiments. The potential of the bioactive phytochemicals derived from the *C. reticulata* seeds shed light on the high capacity of edible fruits’ waste products as a huge reservoir for health-promoting agents.

## 2. Materials and Methods

### 2.1. Plant Material

*C. reticulata* fruits were collected in January 2021 from a local region in Beni-Suef, Egypt, and authenticated by a senior botanist, Prof. Dr. Abd El-Halim A. Mohammed Department of Flora and Phytotaxonomy Research; Horticultural Research Institute; Dokki; Cairo; Egypt). A specimen of the fruits was kept under the voucher number 2021-BuPD 80 at the Pharmacognosy Department; School of Pharmacy; University of Beni-Suef; Egypt).

### 2.2. Chemicals and Reagents

Ethyl acetate (EtOAc), methanol (MeOH), *n*-Hexane (*n*-Hex), ethanol (EtOH), dichloromethane (DCM), and *n*-butanol (*n*-BuOH) were acquired from El-Nasr Company, Egypt. Sigma Aldrich, Saint Louis, MO, USA, provided deuterated solvents for NMR analysis as well as high-purity solvents for HPLC, spectroscopic, and chromatographic analyses (e.g., methanol-d4 (CD_3_OD), dimethyl sulfoxide-d6 (DMSO), chloroform-d (CDCl_3_), methanol for HPLC, HPLC-grade water, and HPLC-grade acetonitrile). For chromatographic isolation, Sephadex LH20 (0.25–0.1 mm, GE Healthcare, Sigma-Aldrich; CA, USA) and Silica gel (E-Merck, Sigma-Aldrich, CA, USA) were used. For vacuum liquid chromatography, a silica gel for TLC (Pharmaceuticals and Chemicals; El-Nasr Company; Cairo, Egypt) was utilized (VLC). Pre-coated silica gel (E-Merck, Darmstadt, Germany) used for TLC studies. Produced spots were identified by *para* anisalde-hyde after heating at 110 °C [7]. All materials for biological investigations came from Sigma Chemical Company (SCC; CA, USA).

### 2.3. Spectral Analyses

NMR signals were obtained at 400 for ^1^H-NMR and 100 MHz for ^13^C-NMR. The CDCl_3_-d, CD_3_OD-*d*_4_, and DMSO-*d*_6_ solvent peaks were employed as reference signals. The NMR signal was acquired using a NMR machine Bruker Advance-III 400 MHz (Bruker AG-Billerica, California, MA, USA).

High resolution mass data were collected using an Acquity-Ultra-Performance-Liquid-Chromatography-machine linked to a Quadrupole-Time-of-Flight-Hybrid-Mass-Spectrometer (Waters, Milford, MA, USA). An Agilent/1260-Infinity-preparative-HPLC was used to achieve HPLC chromatographic separations.

### 2.4. Preparation of C. reticulata Seed Extract

*C. reticulata* seeds (1 kilogram) were purchased fresh and then air-dried in the shade. Following that, the seeds were grated using a grinding machine (Henan, Mainland China). The powdered product was then thoroughly extracted by 70% EtOH (1 L × 5). The resulting liquid extract was concentrated using a Rotary Evaporator (Buchi-Rotavapor-R-300, Cole-Parmer, Vernon Hills, IL, USA) under vacuum at 45 °C to provide 100 g crude dry extract, which was then partitioned using a series of increasing polarity solvents: *n*-Hex-DCM-EtOAc-*n*-BuOH. Fractions I (24.0 g), II (5.0 g), III (3.0 g), and IV (6.0 g) were obtained by evaporating the separated organic phase at decreased pressure. All extracts and fractions were stored at 4 °C for subsequent phytochemical and biochemical analysis.

### 2.5. Isolation of C. reticulata Seeds’ Major Natural Products

TLC-silica gel column was used to expose fraction I (20 g) to normal VLC. EtOAc and *n*-Hex successive elution (0–100%, 5% increasing) were used. Three sub-fractions (I1–I3) were obtained by gathering identical fractions and evaporating them under reduced pressure. On another silica gel column, subfraction I1 (1.0 g) was extensively fractionated. Chromatographic elution was carried out by *n*-Hex to EtOAc, gradient mixes (0–10%, 1% rising, 100 mL each) to provide compound 13. (10 mg).

Sub-fraction I_2_ (50 mg) was further chromatographed on silica-gel-60 (100 × 1 cm, 20 g). Chromatographic procedure was done utilizing DCM:MeOH, isocratic mixture (0.5%, 500 mL) to offer compounds **8** (20 mg) and **9** (15 mg). Sub-fraction I_3_ was somewhat fractionated on silica gel 60 (100 × 1 cm, 20 g). Elution produced employing DCM: MeOH, isocratic mixture (0.5%, 500 mL) to afford **10** (20 mg).

TLC silica-gel column was used to expose fraction II (2.5 g). DCM: EtOAc (0–100%, rising by 5%) was used to elute the samples. The produced effluents were recieved in 250 mL parts. Identical fractions were obtained and reduced to two sub-fractions (II1–II2) at reduced pressure. On another silica gel column subfraction I1 (1.0 g) was extensively fractionated. Compound 5 was eluted using DCM: EtOAc (0–10%, 1 percent rising, 100 mL each) (10 mg).

Sub-fraction I_2_ (50 mg) was further chromatographed on silica gel_60_ (100 × 1 cm, 20 g). Chromatographic elution was applied utilizing DCM to MeOH, (isocratic elution; 0.5%, 500 mL) to offer compounds **11** (20 mg) and **12** (15 mg).

On normal VLC, fraction IV (6.0 g) was separated using silica gel. DCM: EtOAc (0–100%, 5 percent rising, 250 mL each) was used to elute the samples. The chromatographic effluents were recieved in 250 mL increments.

Identical fractions were grouped and reduced under reduced pressure to yield five sub-fractions (IV1–V5); sub-fractions IV1, IV2, IV3, and IV4 were only slightly chromatographed on a sephadex-LH20 column eluted with MeOH to yield **4** (30 mg), **6** (50 mg), **7** (70 mg), and **1** (15 mg, respectively, while sub-fraction IV5 was significantly pur (10 mg).

### 2.6. In Silico Study

#### 2.6.1. Inverse Docking

The probable molecular targets for the separated flavonoids were concluded by subjecting their structures to a virtual screening depends on inverse docking against almost all proteins handed out in the Protein-Data-Bank; PDB; https://www.rcsb.org/; Accessed on 13 January 2022.

The IdTarget online-based platform was employed for this step. This virtual structure-paltry screening program employs a particular inverse docking method, namely Divide-Conquer-Docking, that constructs limited overlapping networks, making the protein’s exploring area more constrained. As a result, it can perform a large number of dockings in a short amount of time [8]. The resulted sores were described as a list of ratings ranging from the worst to the best. To identify the optimal protein targets for each given structure, we used an affinity score of −10 kcal/mol as a cut-off value (Table 1).

#### 2.6.2. Molecular Dynamics Simulation Experiments

Molecular dynamics simulation (MDS) experiments were carried out as earlier reported by Alhadrami et al. 2021 [6]. The MDS machine of Maestro software (i.e., Desmond v. 2.2; Hamburg, Germany) was employed for operating MDS experiments [9,10,11]. The OPLS-Force-Field was utilized for simulation runs.

The protein-structures preparation was performed by the System-Builder algorithm, in which they were packed inside an orthorhombic water box (20 Å^3^) using the TIP3P water model jointly with Na^+^, and Cl^−^ ions, 0.15 M for each of them. Then, the optimized modeled structures were subjected to a preliminary equilibration for 10 ns. Ligand structures were automatically-parameterized during the structure preparation step according to the OPLS-Force-Field. Metal-containing proteins like MMPs that involve histidine-Zn^+2^ complex in the effective site should be parameterized during the protein formation step. To go on, a hetero state should be set up for hetero atoms like Zn (Generate Hetero States). This function is a part of the maestro’s Protein Formation wizard. This step will facilitate the development of a proper hetero state or co-ordinate covalent state, for the heteroatom (i.e., Zn^+2^) in a network with the protein so that force fields like OPLS can quickly observe the zinc atom. For reproductions done by Nano-scale Molecular Dynamics software (NAMD 3.0) [12], the topologies and parameters of modeled structures were determined by the online platform Ligand Reader and Modeler, http://www.charmm-gui.org/?doc=input/ligandrm; accessed on 16 April 2021 [13]. Harmonic Tcl forces were employed to hold Zn^+2^ in place.

#### 2.6.3. Absolute Binding Free Energy Estimation

The calculation of binding free energy (Δ*G*_binding_) was performed as previously described by Alhadrami et al. 2021 [6]. Δ*G*_binding_ were performed using the free energy perturbation (FEP) procedure [12], which is detailed in Kim et al’s publication. In a nutshell, this technique calculates the required accessible energy Δ*G*_binding_ using the equation Δ*G*_binding_ = Δ*G*_Complex_ − Δ*G*_Ligand_. The value of each Δ*G*_binding_ is calculated using the NAMD program on a separate experiments.

All input files can be prepared via the website CharmmGUI: https://charmmgui.org/?doc=input/afes.abinding; accessed on 19 January 2022. The files were then used to produce the planned Δ*G*_binding_ calculations utilizing the FEP protocol in NAMD. The equilibration was later set in the NPT (300 K, 1 atm, Langevin-Piston-Pressure “Complex”-“Ligand”) in the existence of the TIP3P water model. Later, 10 ns FEP simulations were produced for each structure, and the last 5 ns of the available energy amounts was calculated for the finished free energy rates [12]. Ultimately, the binding poses were anticipated and evaluated by employing the VMD program [13].

### 2.7. In Vitro Assay of Hyaluronidase Activity

The activity of hyaluronidase inhibitory were formed according to the earlier established procedure granting to Chaiyana et al. 2019 [14]. The inhibitory effect towards hyaluronidase activity were estimated from the following equation: hyaluronidase inhibition (%) = (1 − A/B) × 100, where A and B are the hyaluronic acid character after treatment in the existence or lack of the sample, separately. As a positive control, 6-*O*-palmitoyl-*L*-ascorbic acid was applied. The experiment was done in triplicate.

### 2.8. In Vitro Assay of Xanthine Oxidase Activity

Xanthine oxidase (XO) inhibitory potential was carried out according to the previously described method [15]. The color produced because of hydrogen peroxide release during the process of oxidation of XO was measured at 570 nm using the plate reader EPOCH™ “MICROPLATE READER (BIOTEK)”. Forty-four microliters of xanthine oxidase buffer were mixed with 2 µL of xanthine substrate solution and 2 µL of XO. L-mimosine was applied as a positive control. This experiment was performed in triplicate.

### 2.9. In Vitro Assay of Tyrosinase Activity

The activity of tyrosinase inhibitory of each flavonoid was observed utilizing the DOPAchrome process conforming to a scheme of Momtaz et al. 2008 [16], which was modified from Nerya et al. 2003 [17], and Curto et al. 1999 [18]. The tyrosinase inhibitory activity was measured from the following equation: inhibitory activity (%) = (1 − A/B) × 100, (9) where A and B are the absorbance of the mixture in presence or absence of test flavonoid, respectively. Every experiment was done in triplicate.

## 3. Results and Discussion

### 3.1. Phytochemical Investigation of C. reticulata Seeds

**Figure 1:**
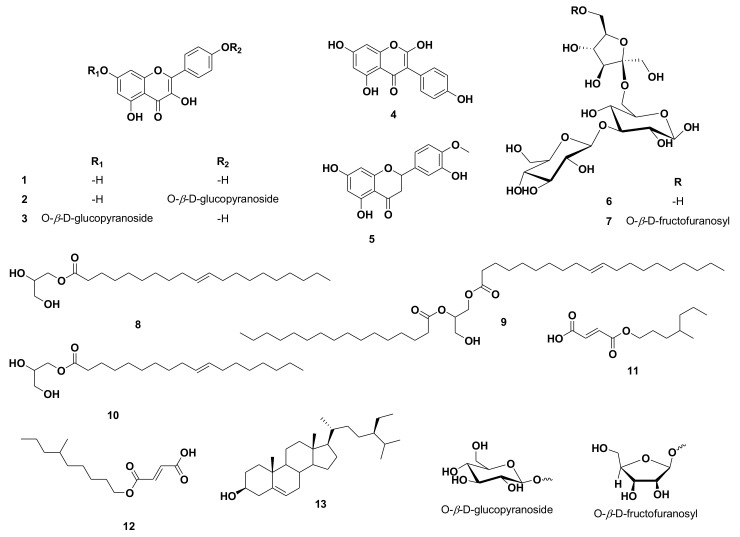
Structures of compounds isolated from *Citrus reticulata* seeds.

Using UV, 1H, and DEPT-Q NMR, beside comparison with the literature and using some authentic samples, the known compounds kaempferol **1** [19], kaempferol-4′-*O*-*β*-*D*-glucopyranoside **2** [19], kaempferol-7-*O*-*β*-*D*-glucopyranoside **3** [19], 2-hydroxygenistein **4** [19], hesperetin **5** [19], 3‴ (1‴-*O*-*β*-*D*-glucopyranosyl)-sucrose **6** [20], 6(1‴-*O*-*β*-*D*-fructofuranosy)-3′ (1″-*O*-*β*-*D*-glucopyranosyl)-sucrose **7** [20], 1-*O*-elaidoyl-glycerol **8** [21], 1-*O*-oleoyl-2-palmitoyl-glycerol **9** [22], 1-*O*-oleoyl-glycerol **10** [21], (*E*)-3-((4-methylheptyloxy)carbonyl)acrylic acid **11** [23], (E)-3-((6-methylnonyloxy)carbonyl)acrylic acid **12** [23] and *β*-sitosterol **13** [24] (Figure 1) were identified from the crude ethanolic extract of *C. reticulata* seeds. All identified compounds **11** and **12** were separated herein for the first time from the *Citrus* genus (Figures S1–S28 and Figure 1). It is worth noting that unlike the widely distributed flavanones and flavones in *C. reticulata* aerial parts, including the fruits, flavonols appeared to be the predominant type of flavonoids in the seeds.

### 3.2. In Silico Investigation

#### Inverse Docking

To characterize the biological activity of the isolated compounds, we subjected them to a series of in silico-based analyses. First, we focused on the isolated flavonoids (**1**–**5**) for our initial virtual screening step because flavonoids are well-known bioactive plant secondary metabolites. Additionally, the remaining isolated compounds (**6**–**13**) are common primary metabolites that likely have no therapeutic efficacy.

The structures of flavonoids **1**–**5** were subjected to a docking-based virtual screening against almost all proteins hosted in PDB. The online platform, namely idTarget was employed for this preliminary virtual screening task [8]. The recovered scores were retrieved as a list from the largest negative value to the smallest one. We set a conclusive affinity score of −10 kcal/mol as a cut-out value to single out the good targets for each isolated flavonoid (1–5). Several interesting molecular targets were found out to be a probable good target for these related compounds, including protein kinases and synthases (Table 1).

Interestingly, three common molecular targets were reported between these flavonoids, hyaluronidase, xanthine oxidase and tyrosinase enzymes. These enzymes are related to maintaining healthy skin [25,26,27,28,29,30], and hence the preliminary virtual screening step putatively identified these flavonoids as probable skin care promoting agents.

### 3.3. Molecular Dynamics Simulation and Absolute Binding Free Energy Calculation

To further support the preliminary docking-based screening step, we estimated the absolute binding free energies (Δ*G*_binding_) of compounds **1**–**5** with hyaluronidase, xanthine oxidase and tyrosinase enzymes using the MDS-dependent method, namely the free energy perturbation method (FEP) [26,31].

As shown in Table 2, only compounds **2**, **3** and **5** that were able to achieve the highest affinity towards hyaluronidase, xanthine oxidase and tyrosinase enzymes with Δ*G*_binding_ values ranged from −8.1 to −9.9 kcal/mol. The remaining compounds got Δ*G*_binding_ values higher than −6 kcal/mol.

**Figure 2:**
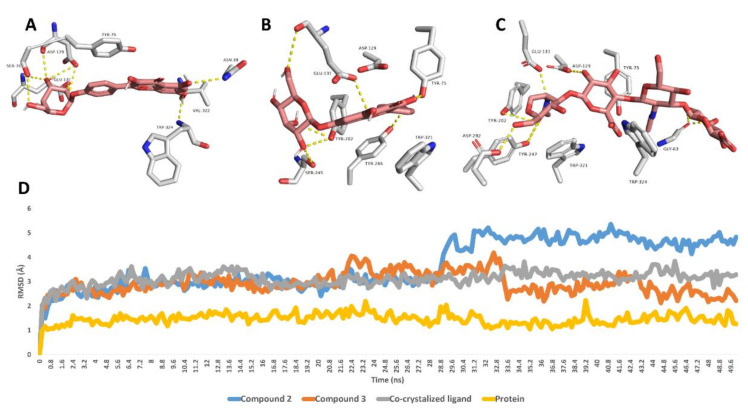
Binding modes of compounds **2**, **3** and the co-crystalized ligand inside the active site of hyaluronidase derived from the last snapshot of their 50 ns MDS (**A**–**C**). RMSDs of compounds **2**, **3** and the co-crystalized ligand inside the active site of hyaluronidase over the 50 ns MDS (**D**).

Further 50 ns MDS experiments were performed to investigate the interactions and binding stability of compounds **2**, **3** and **5** with hyaluronidase, xanthine oxidase and tyrosinase enzymes. As depicted in Figure 2, both compound **3** and the co-crystalized ligand achieved similar binding stability inside the enzyme’s binding site over the course of MDS with RMSD of ~2.8 Å from the initial docking poses. Compound **2′**s RMSDs were also like that of compound **3** until 28.7 ns, when it begins to deviate slightly higher from compound **3** and the co-crystalized ligand to achieve an average RMSD of 3.8 Å over the course of MDS. The last snapshot derived from the MDS of compounds **2**, **3** and the co-crystalized ligand indicated that TYR-75, ASP-129, GLU-131 were the common interacting amino acid residues via H-bonding.

**Figure 3:**
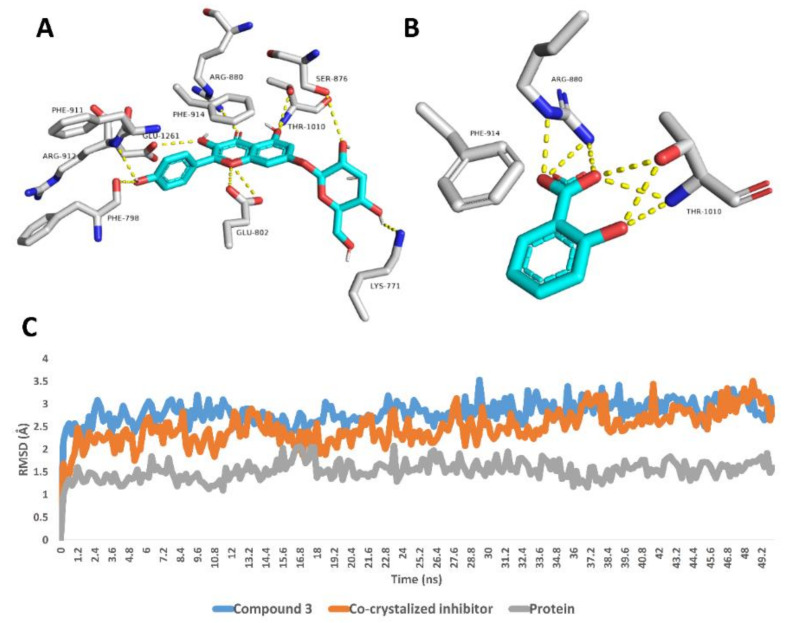
Binding modes of compound **3** and the co-crystalized inhibitor inside the active site of xanthine oxidase derived from the last snapshot of their 50 ns MDS (**A**,**B**). RMSDs of compound **3** and the co-crystalized inhibitor inside the active site of xanthine oxidase over the 50 ns MDS (**C**).

Regarding xanthine oxidase, compound **3** achieved stable binding inside its active site over the course of MDS, recording an average RMSD of 2.6 Å that was convergent to that of the co-crystalized inhibitor (RMSD ~2.4 Å). Such stable binding of compound **3** was obviously due to the H-bonds network that it established with LYS-771, PHE-798, GLU-802, SER-876, ARG-880, PHE-911, ARG-912 and THR-1010 (Figure 3).

**Figure 4:**
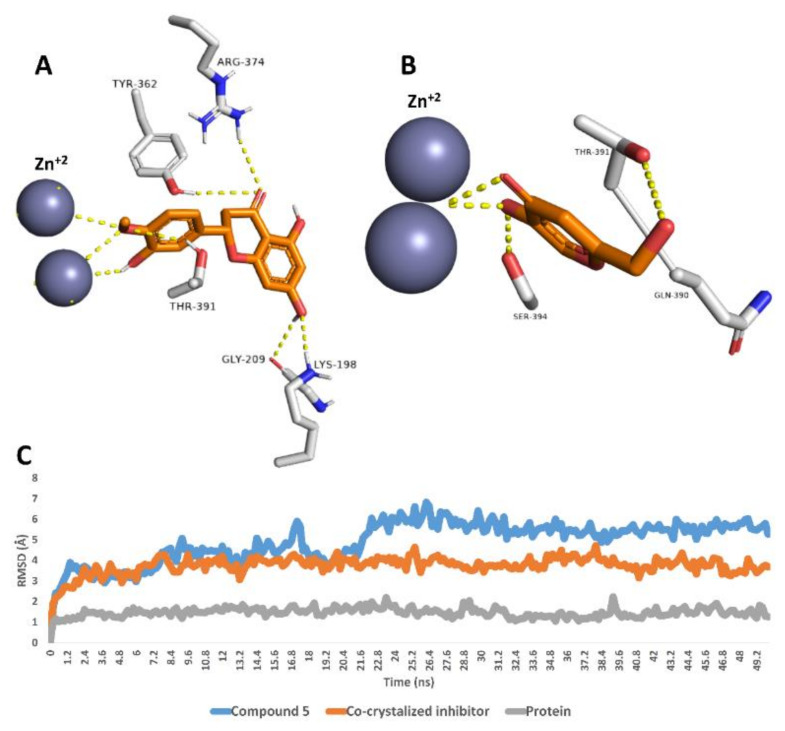
Binding modes of compound **5** and the co-crystalized inhibitor inside the active site of tyrosinase derived from the last snapshot of their 50 ns MDS (**A**,**B**). RMSDs of compounds 5 and the co-crystalized inhibitor inside the active site of tyrosinase over the 50 ns MDS (**C**).

Finally, compound **5** was also stable inside the tyrosinase’s active site over the course of 50 ns MDS with an average RMSD value of 4.9 Å, which was higher than that of the co-crystalized inhibitor (RMSD ~3.8 Å). As shown in the last MDS-derived snapshot, compound 5 was able to interact with the two Zn^2+^ ions similarly to the co-crystalized inhibitor. In addition, it was stabilized with several H-bonds with LYS-198, GLY-209, TYR-362, ARG-374 and THR-391 (Figure 4).

### 3.4. In Vitro Assays

To validate the previously discussed in silico findings, we tested compounds **2**, **3** and **5** for their inhibitory activity against hyaluronidase, xanthine oxidase and tyrosinase enzymes in vitro. As presented in Table 3, compound **3** was identified as a potent hyaluronidase inhibitor followed by compound **2** with IC_50_ values of 9.5 ± 0.48 and 13.7 ± 1.08 µM, respectively. The known 6-*O*-palmitoyl-L-ascorbic acid (IC_50_ 2.033 ± 0.1 µM) was used as a positive control. Compound **5** was inactive against hyaluronidase. Regarding xanthine oxidase, compound **3** was significantly able to inhibit its activity with the IC_50_ value of 6.39 ± 0.36 µM, while both compound **2** and **5** showed weak or inactivity, the known L-mimosine (IC_50_ 3.63 ± 0.18 µM) was used as a positive control. Finally, compound 5 was the most active compound against tyrosinase, with an IC_50_ value of 8.67 ± 0.44 µM, while compounds **2** and **3** were inactive or weakly active, and the known kojic acid (IC_50_ 6.52 ± 0.33 µM) was used as a positive control. These in vitro results revealed the potential of *C. reticulata* seed-derived flavonoids, particularly compounds **2**, **3** and **5**, as healthy skin promoting agents via their inhibition of the activity of a number of relevant enzymes (i.e., hyaluronidase, xanthine oxidase and tyrosinase enzymes). Additionally, they showed the applicability of using different in silico-based analysis as a preliminary screening step in the characterization of biological activity in natural products.

Extracellular matrix (ECM) degradation is the primary cause of skin aging [32]. Collagenase and gelatinases (MMP-2) are matrix metalloproteinases (MMPs) that have a role in ECM degradation [33]. As a result, the skin's tensile strength is depleted. Roughness, wrinkling, and dehydration of the skin still occur often, as do various pigment anomalies such as hy-po-/hyper-pigmentation [32,34]. Tyrosinase inhibitors have been studied for the treatment of hyperpigmentation of the skin.

The enzyme tyrosinase converts tyrosine to melanin [35]. As a result, tyrosine inhibitors play an important role as skin-lightening agents [36]. Hyaluronic acid (HA) production is being studied for the treatment of skin wrinkles. The presence of wrinkles and skin moisture have both been linked to HA. HA also deals with tissue improvement, including immune system response augmentation through inflammatory cell activation and fibroblast injury [37,38]. Hyaluronidase is a proteolytic enzyme found in the dermis that is responsible for the breakdown of hyaluronan in the extracellular matrix, resulting in visible signs of skin aging [39].

As a result, hyaluronidase inhibitors are crucial in the treatment of skin wrinkles. XO is also a major source of oxidants and plays a role in a number of oxidative stress-related diseases. Because of the ongoing oxidative stress situation, aging is associated with a progressive deregula-tion of homeostasis [40]. As a result, XO inhibitors affect skin aging treatment.

The findings of this study showed that *C. reticulata* seed-derived flavonoids, particularly compounds 2, 3 and 5, can promote healthy skin by inhibiting the activity of hyaluronidase, xanthine oxidase, and tyrosinase enzymes. Compound **3** was found to be a potent hyaluronidase inhibitor, followed by compound 2 with IC_50_ values of 9.5 0.48 and 13.7 1.08 M, respectively. With an IC_50_ value of 6.39 0.36 M, compound 3 was able to strongly inhibit the activity of xanthine oxidase. With an IC_50_ value of 8.67 0.44 M, compound 5 was the most potent chemical against tyrosinase (Table 3).

The societal, therapeutic, and commercial difficulties posed by non-healing wounds are growing as our society ages. As a result, studying the impact of aging on wound healing has become a popular issue [41]. Skin functions deteriorate with age due to anatomical and morphological changes directed by innate factors such as historical make-up, changes in hormone stages, and exogenous factors such as sun exposure and cigarette smoking [42]. Aging skin changes not only affect wound healing but also make the skin particularly susceptible to wounds. Devaluation of nerve endings, for example, diminishes pain sensitivity, increasing the risk of damage, and epidermal degeneration causes the skin to become more susceptible to mechanical forces.

The growth of chronic wounds is aided by immunosenescence. Microvascular disruptions may also reveal the fate of ischemic lesions [41,42].

Flavonoids are found in abundance as bioactive secondary metabolites. They are found in a variety of medicinal plants that are used to improve wound healing [43]. Topical application of kaempferol 1, which has anti-inflammatory and antioxidant properties, was found to have healing effects on incisional and excisional wounds in diabetic and non-diabetic rats [44]. Kaempferol 1 mediated these effects by increasing wound collagen and hydroxyproline output, improving wound protection, speeding wound closure, and hastening re-epithelialization.

Furthermore, kaempferol and its glycosides derivatives 2–3 exhibited astringent and antimicrobial properties that were found to be useful for wound shrinkage and enhancing the rate of epithelialization in male Wistar rats using an excision and incision wound model, as well as encouraging the movement of CCD-1064sk fibroblasts into a scratch wound assay on Ha-CaT keratinocytes [45,46]. Moreover, isoflavonoid (e.g., 2-hydroxygenistein, 4) has been shown to promote wound healing by increasing tensile strength, reducing inflammation, and inhibiting collagenase, hyaluronidase, and elastase enzymes [47].

Genistein, a 2-deoxy derivative of 2-hydroxygenistein 4, has been linked to soy's beneficial effects, particularly in the context of ageing. Cut intrinsic estrogen leads to a range of age-related diseases in postmenopausal women, including protracted cutaneous wound healing. Genistein accelerated wound healing while suppressing the inflammatory response. Genistein’s actions were limited to interfering with estrogen receptor-dependent signaling [48]. In sham OVX rats, genistein reduced tissue transglutaminase-2, TGF-1, and vascular endothelial growth factor, indicating that genistein derivatives have anti-ageing aesthetic characteristics [49].

Hesperidin has sufficient healing benefits on injured skin. Hesperidin can thus be utilized as a supplement or alternative to other wound-healing agents [50,51,52]. Aside from flavonoids, fatty acid esters of glycerol [53,54], acrylic acid derivatives [55], and sterols [56] all had similar wound healing effects. The potential of *C. reticulata* seed extract in age-related characteristics of cutaneous wound healing was disclosed in this literature, however more in vivo testing is needed.

## 4. Conclusions

Herein, we investigated the chemical composition of *C. reticulata* seeds via stepwise chromatographic isolation and the subsequent spectroscopic-based structural identification. Flavonols were found to be the most prevalent type of flavonoids in the investigated seeds instead of the well-known predominance of flavanones and flavones in the aerial parts, including the fruits. Additionally, several other common oligosaccharides, sterols and fatty acids were also found to be major metabolites. In silico-based study of the isolated flavonoids aiming at characterizing their pharmacological effects highlighted their potential as hyaluronidase, xanthine oxidase and tyrosinase inhibitors. Further MDS-based investigation selected compounds **2**, **3** and **5** to be the most promising candidates against these skin-related enzymes. Final in vitro enzyme assays revealed the potential of these compounds (i.e., **2**, **3** and **5**) as skin-promoting agents via their inhibitory activity against hyaluronidase, xanthine oxidase and tyrosinase activity. This study highlighted the waste product of *C. reticulata* seeds as a very good source of healthy skin-promoting phytochemicals and age-linked features of cutaneous wound healing. Additionally, it revealed the power of integrating inverse docking with MDS experiments in characterizing the biological activities of natural products.

## Figures and Tables

**Table 1:** Inverse docking results of compounds **1**–**5**. Binding scores were calculated as kcal/mol. A cut-off value of −10 kcal/mol were set to choose the best protein targets for each compound.

Predicted Target	PDB Code	1 (Score)	2 (Score)	3 (Score)	4 (Score)	5 (Score)
**Electron transfer flavoprotein**	1efp	>−10	>−10	−10.2	−10.4	>−10
**Orotidine 5’-phosphate decarboxylase**	2cze	>−10	−10.5	>−10	>−10	−12.2
**Hyaluronidase**	**1fcv**	**−11.4**	**−10.9**	**−12.3**	**−10.6**	**−11.6**
**Protein l-isoaspartate O methyltransferase**	1i1n	>−10	>−10	−12.4	>−10	>−10
**Tyrosinase**	**5 m 8 m**	**−11.1**	**−11.6**	**−11.7**	**−10.3**	**−12.7**
**Threonine synthase**	1v7c	−11.8	−12.1	>−10	>−10	>−10
**Factor7-413 complex**	1w7x	>−10	>−10	>−10	−11.4	>−10
**Alpha-glycerophosphate oxidase**	2rgh	>−10	>−10	>−10	>−10	−10.2
**Xanthine oxidase**	**1fiq**	**−10.7**	**−11.3**	**−10.5**	**−10.9**	**−10.6**
**Cyclopropane synthase**	1tpy	>−10	−11.5	>−10	>−10	>−10
**Phosphoglycerate kinase**	2wzc	−12.5	>−10	>−10	>−10	>−10
**S-adenosyl-l-methionine-dependent methyltransferase**	3dlc	>−10	>−10	>−10	−11.1	>−10
**Factor viia-tissue factor**	1wun	>−10	>−10	−12.8	>−10	>−10
**Cyclin-dependent kinase 6**	1xo2	>−10	−12.3	>−10	>−10	>−10
**3-o-sulfotransferase-3**	1t8u	−10.4	>−10	>−10	>−10	−11.8
**Glycolate oxidase**	1gox	>−10	>−10	−10.4	>−10	−10.2
**6,7-dimethyl-8-ribityllumazine synthase**	2a59	>−10	>−10	>−10	>−10	−10.4
**Nicotinamide riboside kinase**	2ql6	>−10	>−10	>−10	−10.3	>−10

**Table 2:** Docking scores and calculated Δ*G*_binding_ values (expressed as kcal/mol) of compounds **1**–**5** with hyaluronidase, xanthine oxidase and tyrosinase enzymes.

Structures	Hyaluronidase (PDB:1FCV)		Xanthine Oxidase (PDB: 1FIQ)		Tyrosinase (PDB: 5M8M)	
	Docking Score	Δ*G*_binding_	Docking Score	Δ*G*_binding_	Docking Score	Δ*G*_binding_
**1**	−11.4	−5.6	−10.7	−5.9	−11.1	−5.3
**2**	−10.9	**−8.9**	−11.3	−5.6	−11.6	−4.5
**3**	−12.3	**−9.9**	−10.5	**−8.9**	−11.7	−5.8
**4**	−10.6	−5.5	−10.9	−4.8	−10.3	−5.6
**5**	−11.6	−5.4	−10.6	−5.1	−12.7	**−8.1**

**Table 3:** In vitro inhibitory potential of compounds **2**, **3** and **5** against hyaluronidase, xanthine oxidase and tyrosinase enzymes (expressed as IC_50_ in µM ± SD).

Compounds	Hyaluronidase	Xanthine Oxidase	Tyrosinase
**2**	13.7 ± 1.08	73.7± 2.33	>100
**3**	9.5 ± 0.48	6.39 ± 0.36	57.2 ± 2.91
**5**	>100	>100	8.67 ± 0.44
**6-*O*-palmitoyl-L-ascorbic acid**	2.033 ± 0.1	-	-
**L-mimosine**	-	3.63 ± 0.18	-
**Kojic Acid**	-	-	6.52 ± 0.33

6-*O*-palmitoyl-L-ascorbic acid, L-mimosine and kojic Acid were used as reference inhibitors for hyaluronidase, xanthine oxidase and tyrosinase enzymes, respectively.

## Data Availability

Data is contained within the article and supplementary material.

## Supplementary Materials

The following supporting information can be downloaded at: https://www.mdpi.com/article/10.3390/antiox11050984/s1, Figure S1: 1H NMR spectrum of compound 1 measured in CD3OD-d4 at 400 MHz; Figure S2: DEPT-Q NMR spectrum of compound 1 measured in CD3OD-d4 at 100 MHz; Figure S3: 1H NMR spectrum of compound 2 measured in CD3OD-d4 at 400 MHz; Figure S4: DEPT-Q NMR spectrum of compound 2 measured in CD3OD-d4 at 100 MHz; Figure S5: 1H NMR spectrum of compound 3 measured in CD3OD-d4 at 400 MHz; Figure S6: DEPT-Q NMR spectrum of compound 3 measured in CD3OD-d4 at 100 MHz.; Figure S7: 1H NMR spectrum of compound 4 measured in CD3OD-d4 at 400 MHz; Figure S8: DEPT-Q NMR spectrum of compound 4 measured in CD3OD-d4 at 100 MHz; Figure S9: 1H NMR spectrum of compound 5 measured in DMSO-d6 at 400 MHz; Figure S10: DEPT-Q NMR spectrum of compound 5 measured in DMSO-d6 at 100 MHz: Figure S11: 1H NMR spectrum of compound 6 measured in CD3OD-d4 at 400 MHz; Figure S12: DEPT-Q NMR spectrum of compound 6 measured in CD3OD-d4 at 100 MHz; Figure S13: HSQC spectrum of compound 6 measured in CD3OD-d4; Figure S14: HMBC spectrum of compound 6 measured in CD3OD-d4; Figure S15: 1H NMR spectrum of compound 7 measured in CD3OD-d4 at 400 MHz; Figure S16: DEPT-Q NMR spectrum of compound 7 measured in CD3OD-d4 at 100 MHz; Figure S17: H NMR spectrum of compound 8 measured in DMSO-d6 400 MHz; Figure S18: DEPT-Q NMR spectrum of compound 8 measured in DMSO-d6 at 100 MHz; Figure S19: 1H NMR spectrum of compound 9 measured in DMSO-d6 at 400 MHz; Figure S20: DEPT-Q NMR spectrum of compound 9 measured in DMSO-d6 at 100 MHz; Figure S21: 1H NMR spectrum of compound 10 measured in DMSO-d6 at 400 MHz; Figure S22: DEPT-Q NMR spectrum of compound 10 measured in DMSO-d6 at 100 MHz; Figure S23: 1H NMR spectrum of compound 11 measured in DMSO-d6 at 400 MHz; Figure S24: DEPT-Q NMR spectrum of compound 11 measured in DMSO-d6 at 100 MHz; Figure S25: 1H NMR spectrum of compound 12 measured in CDCL3-d at 400 MHz; Figure S26: DEPT-Q NMR spectrum of compound 12 measured in CDCL3-d at 100 MHz; Figure S27: 1H NMR spectrum of compound 13 measured in CDCL3-d at 400 MHz; Figure S28: DEPT-Q NMR spectrum of compound 13 measured in CDCL3-d at 100 MHz.
