# Supplementary material for: Bioactive Phytochemicals of Citrus reticulata Seeds—An Example of Waste Product Rich in Healthy Skin Promoting Agents
Source: Antioxidants (Basel). 2022 May 18;11(5):984. doi: 10.3390/antiox11050984 (PMC9138151; doi:10.3390/antiox11050984)
Supplement: Supplementary file 1 [file antioxidants-11-00984-s001.zip › antioxidants-1659802-supplementary.pdf]

Supplementary Materials

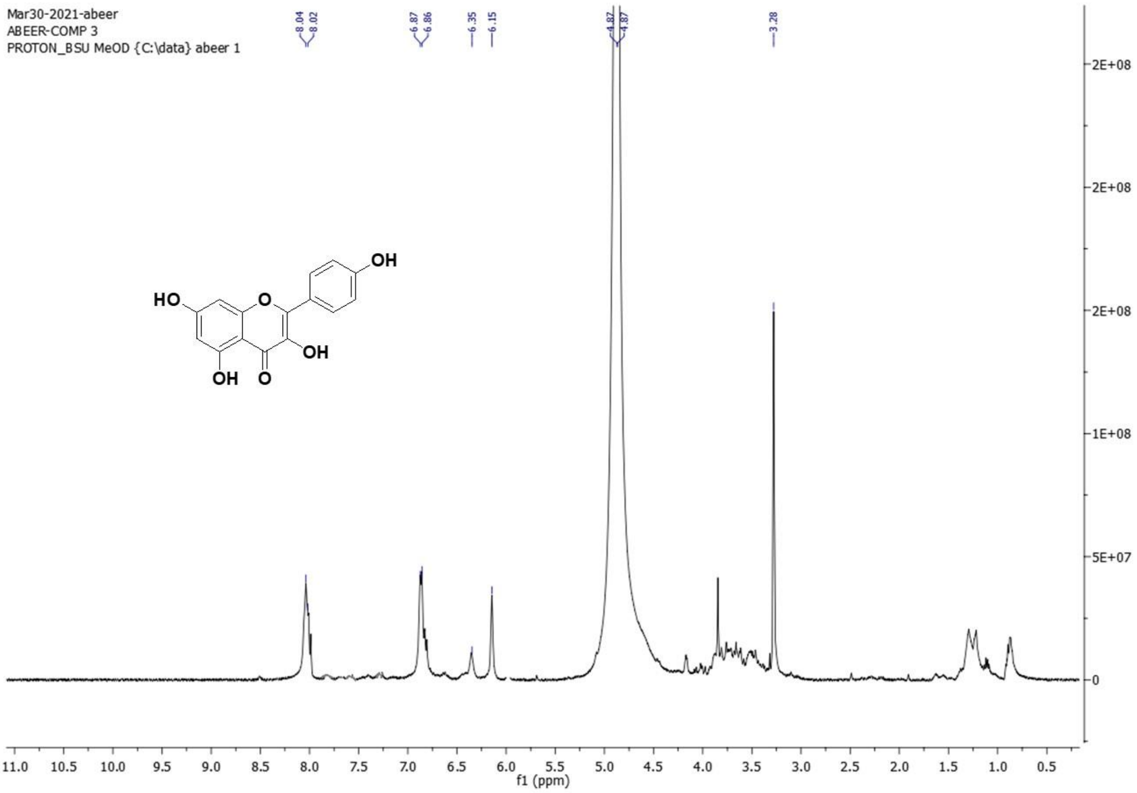

Figure S1. 1H NMR spectrum of compound **1** measured in CD_3OD-*d*$_4$ at 400 MHz.

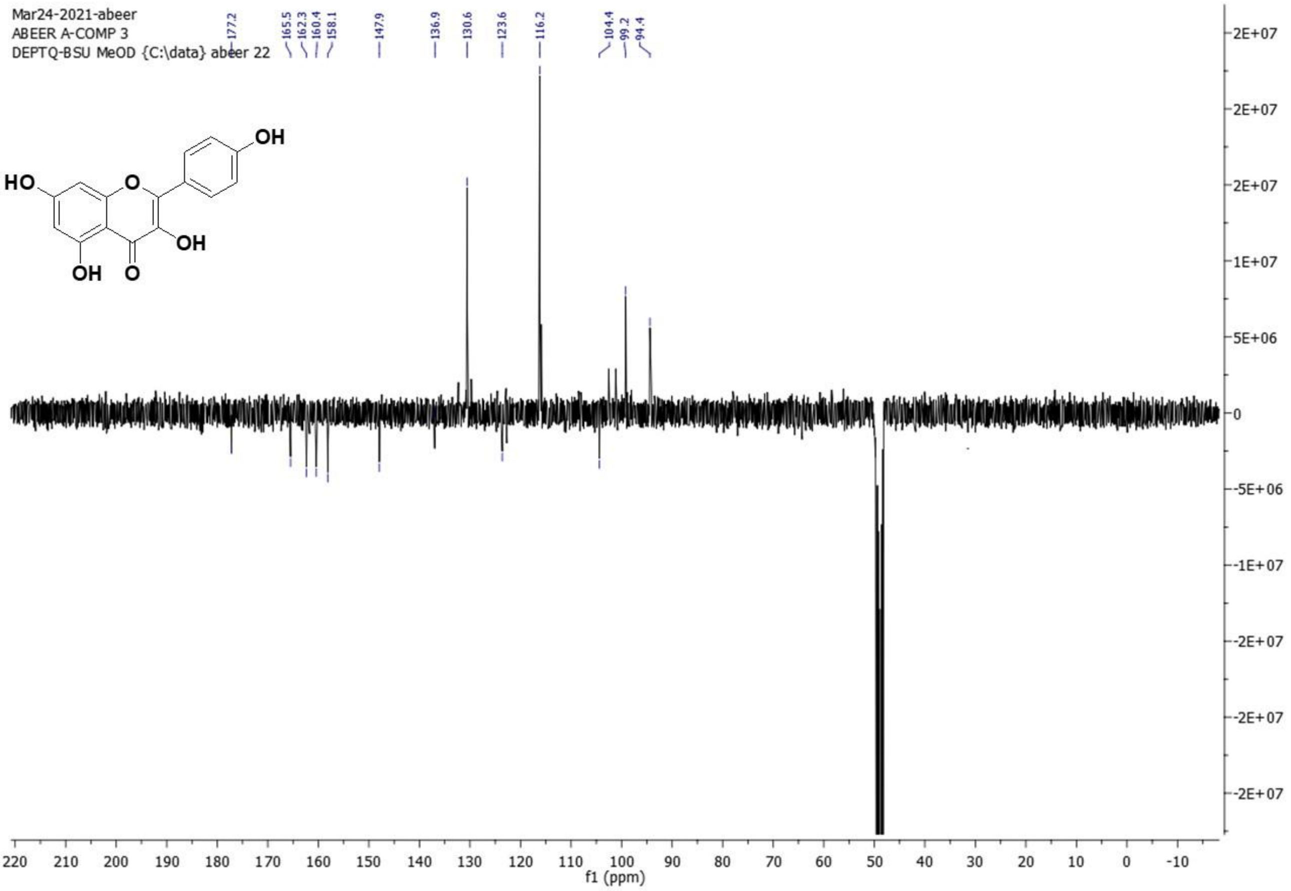

Figure S2. DEPT-Q NMR spectrum of compound **1** measured in CD_3OD-d_4 at 100 MHz.

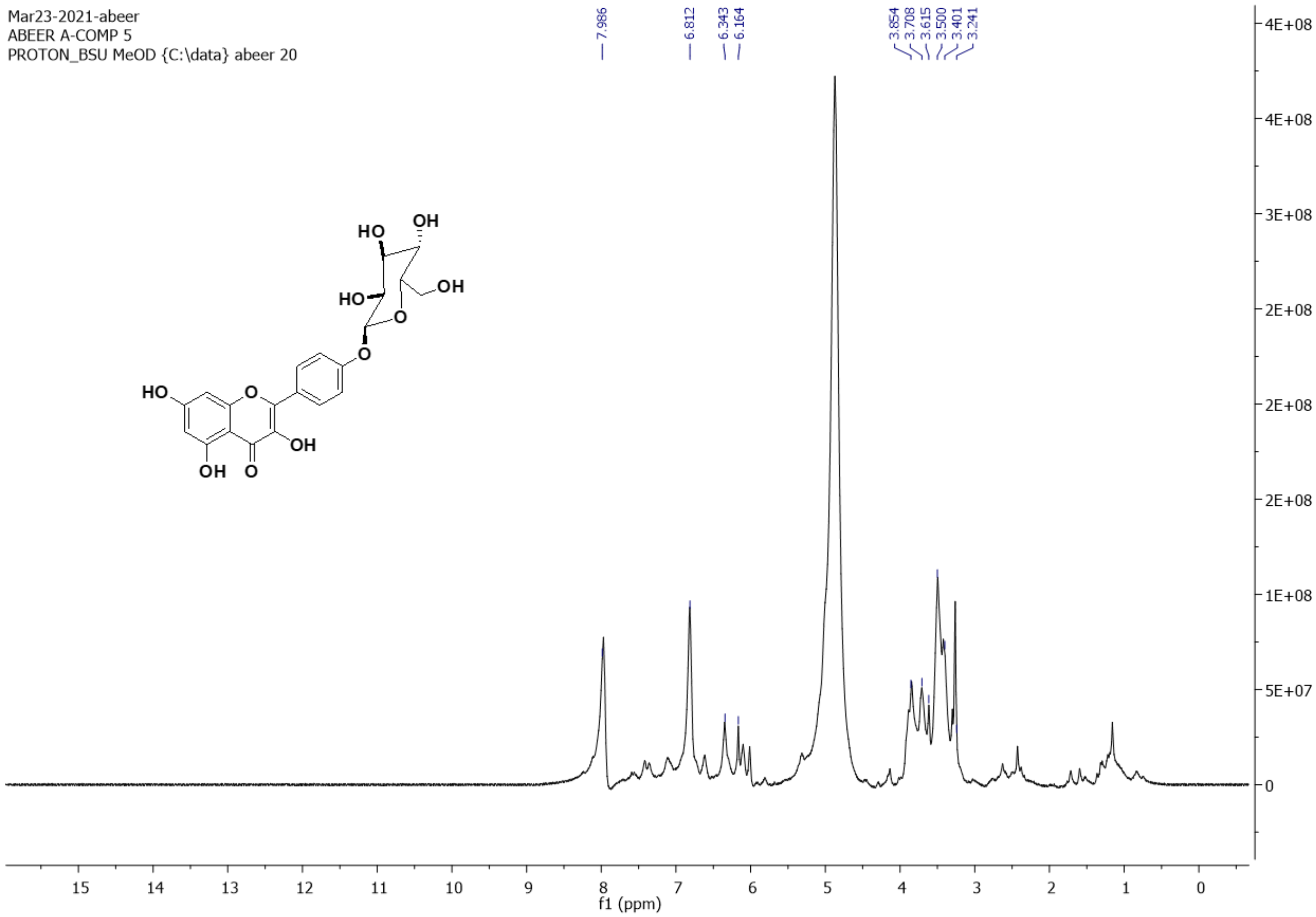

Figure S3. 1H NMR spectrum of compound **2** measured in CD_3OD-d_4 at 400 MHz.

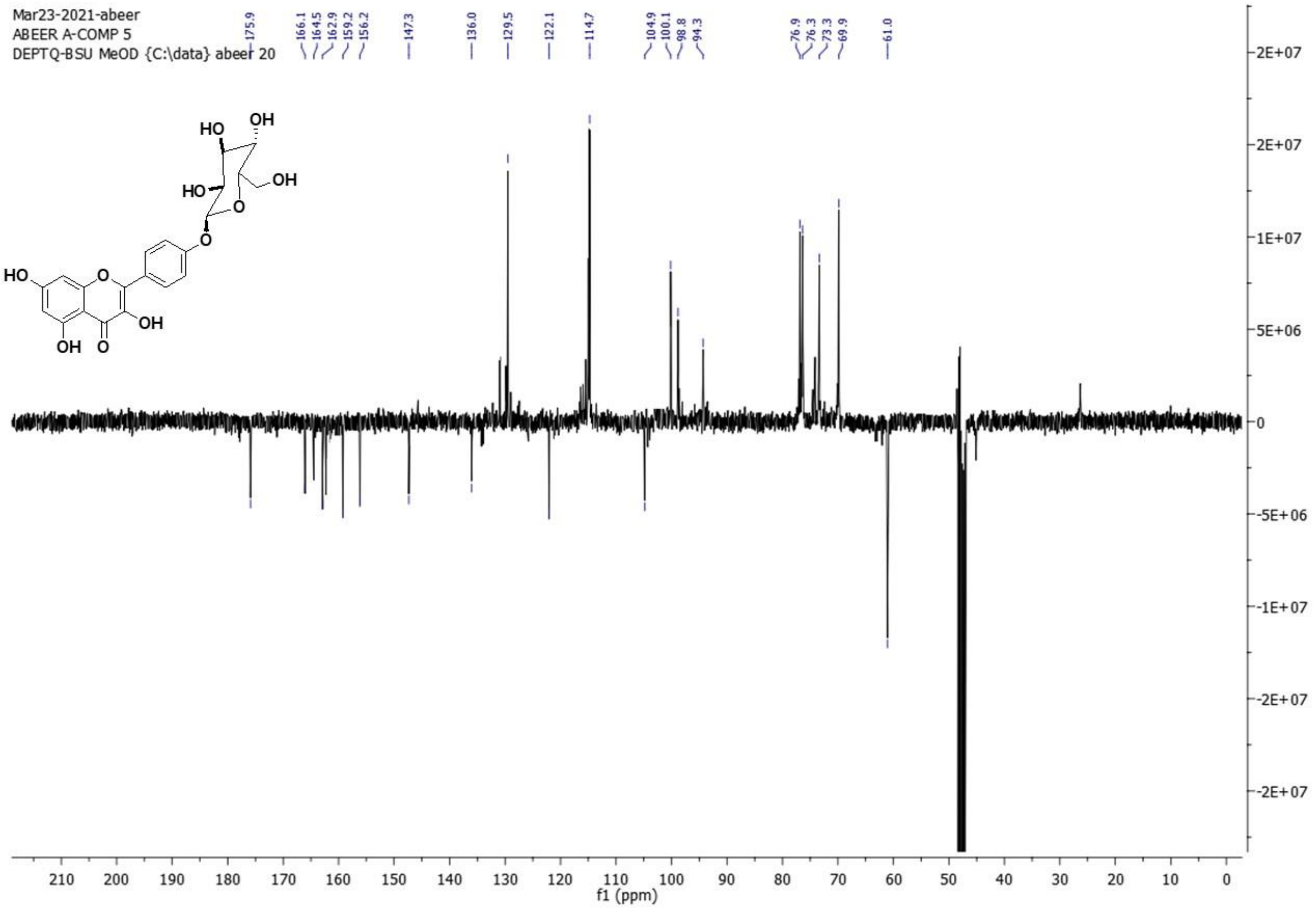

Figure S4. DEPT-Q NMR spectrum of compound **2** measured in CD_3OD-d_4 at 100 MHz.

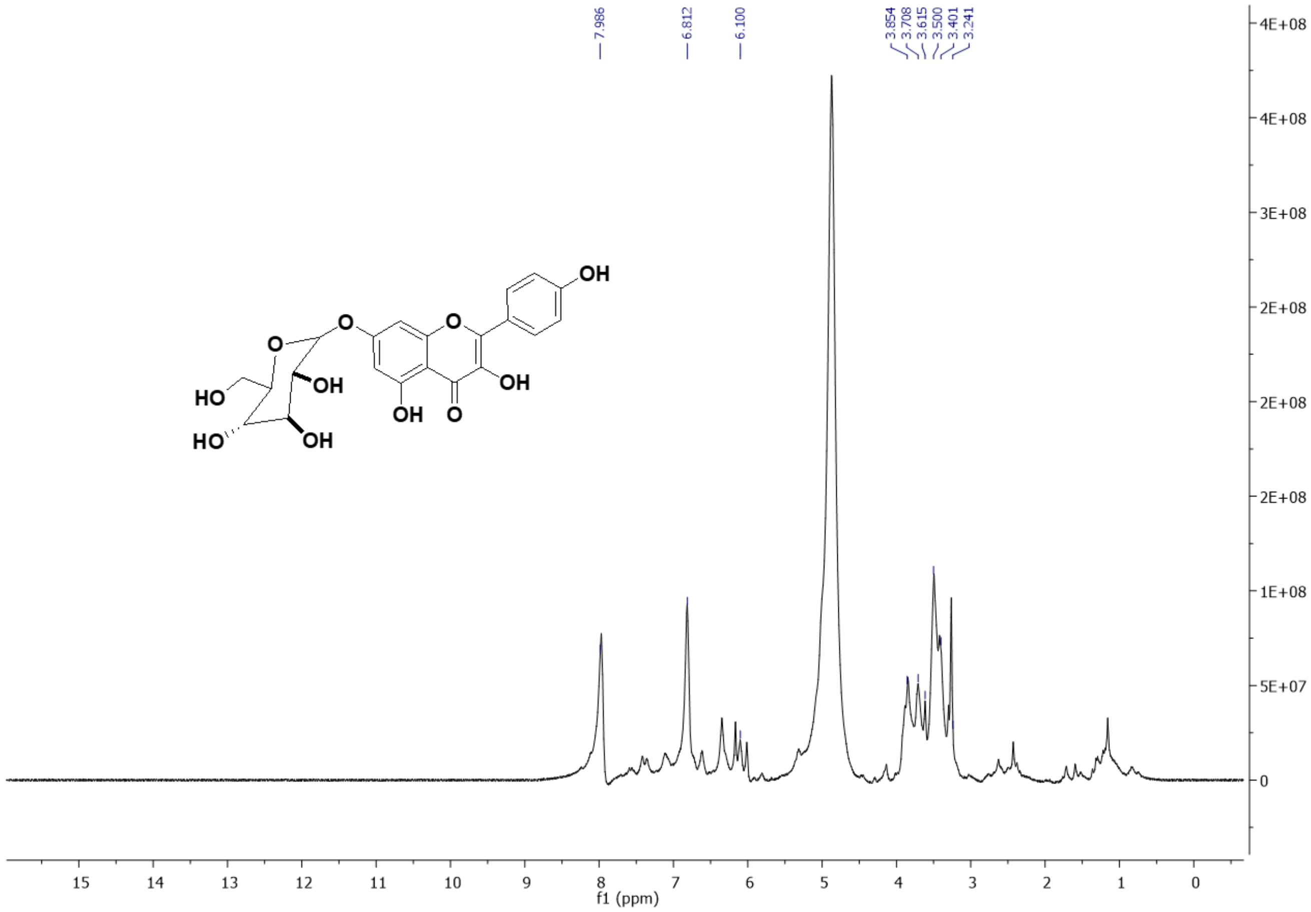

Figure S5. 1H NMR spectrum of compound **3** measured in CD_3OD-*d*$_4$ at 400 MHz.

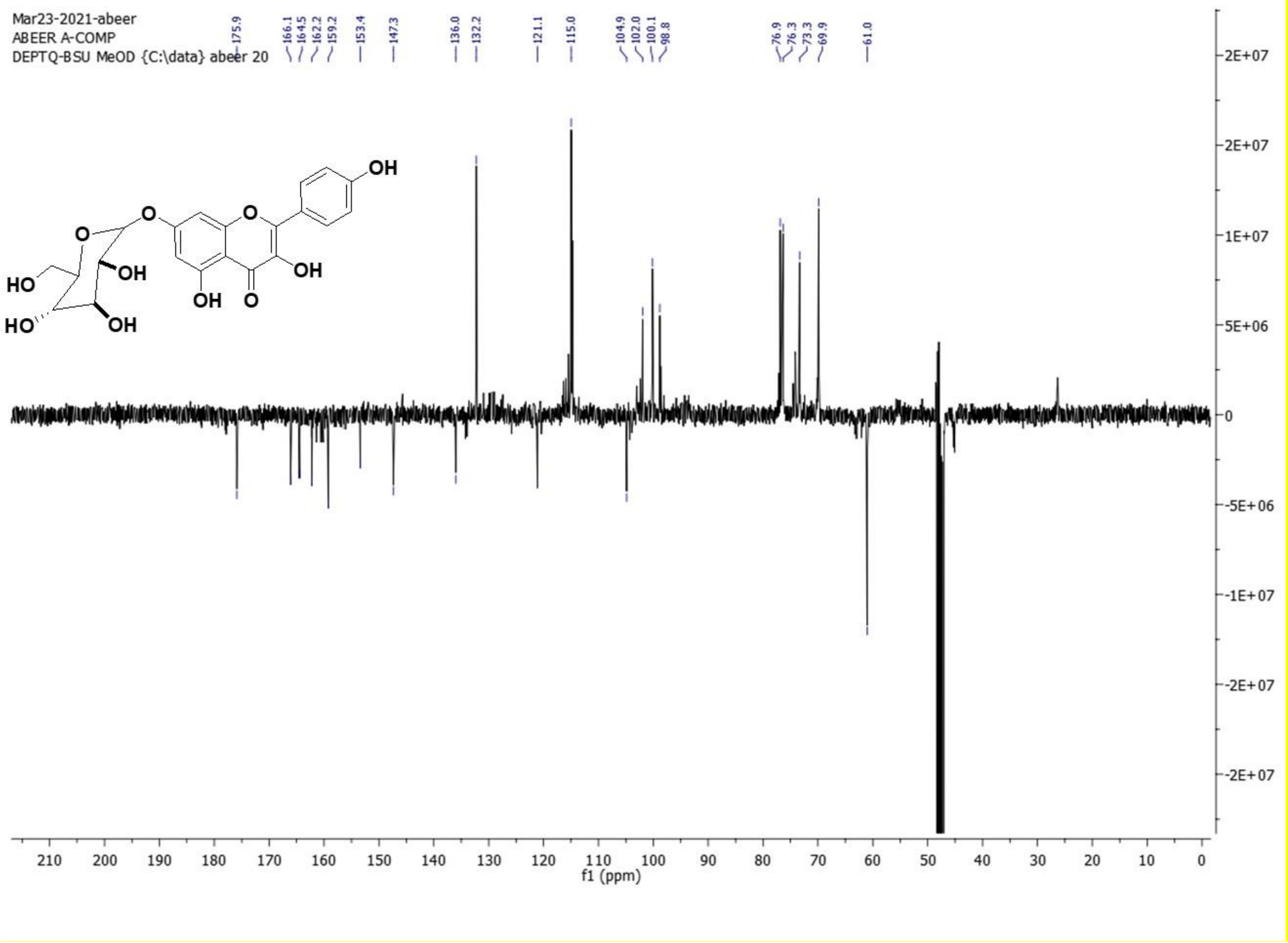

Figure S6. DEPT-Q NMR spectrum of compound **3** measured in CD_3OD-d_4 at 100 MHz.

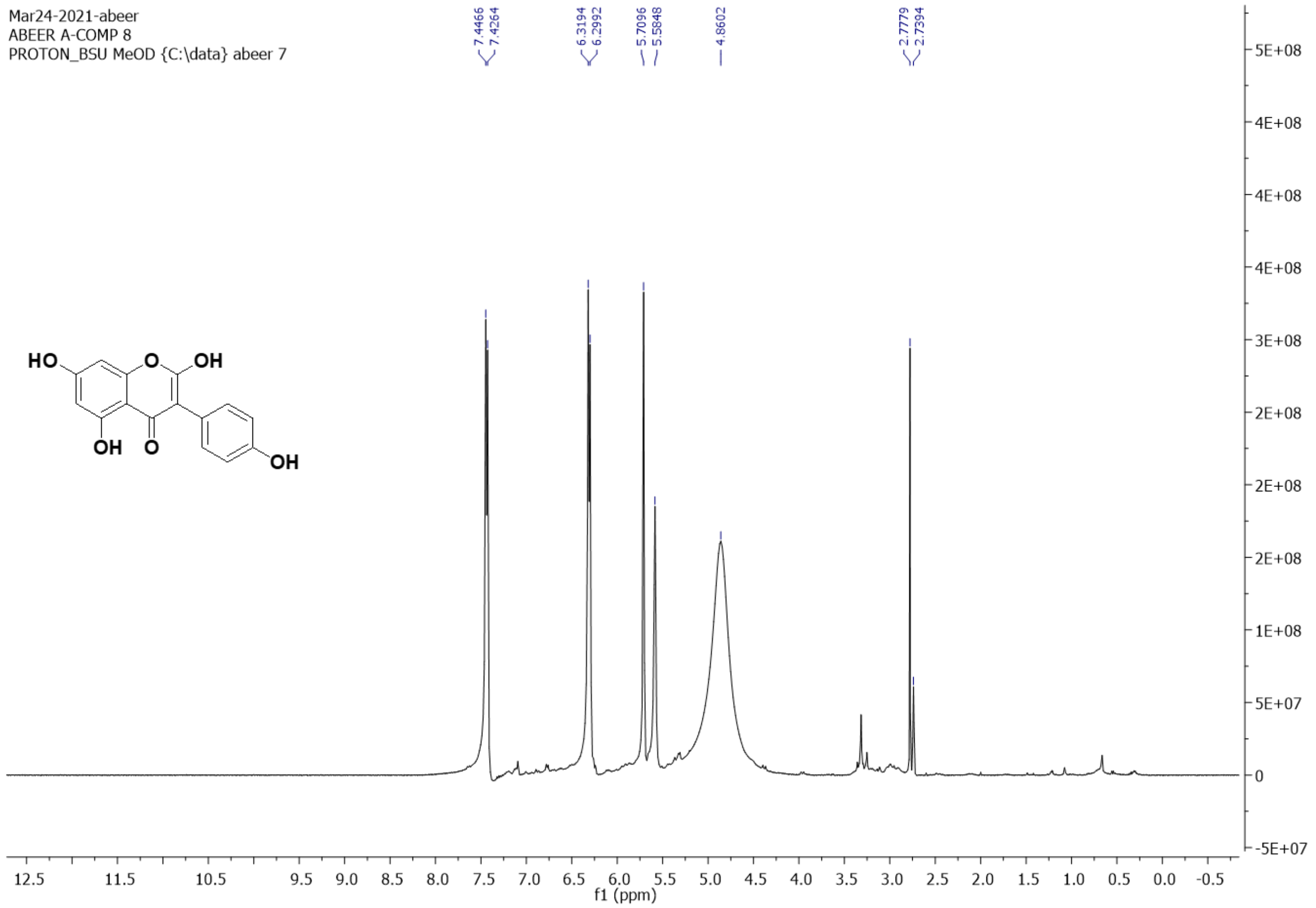

Figure S7. 1H NMR spectrum of compound **4** measured in CD_3OD-d_4 at 400 MHz.

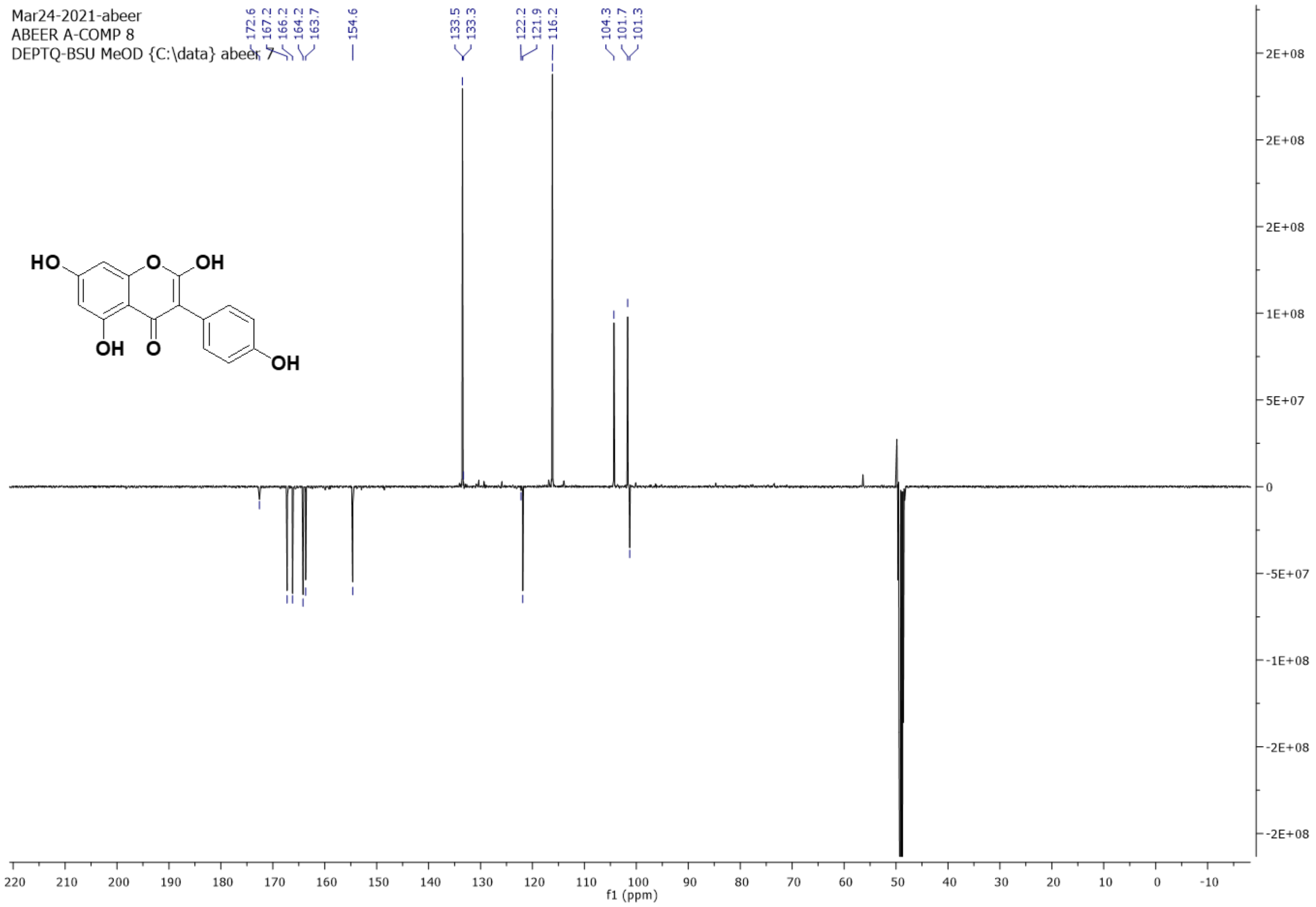

Figure S8. DEPT-Q NMR spectrum of compound **4** measured in CD_3OD-d_4 at 100 MHz.

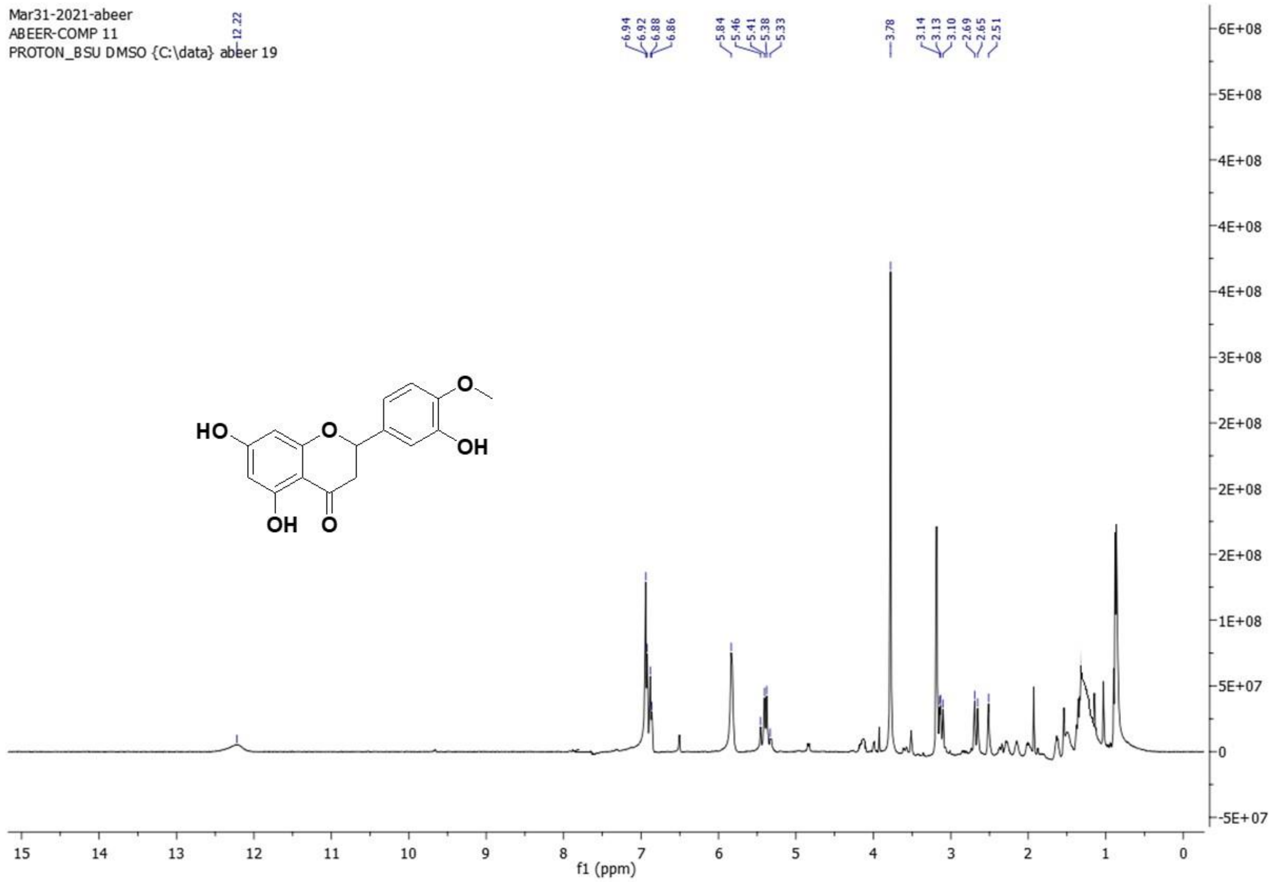

Figure S9. 1H NMR spectrum of compound **5** measured in DMSO-d_6 at 400 MHz.

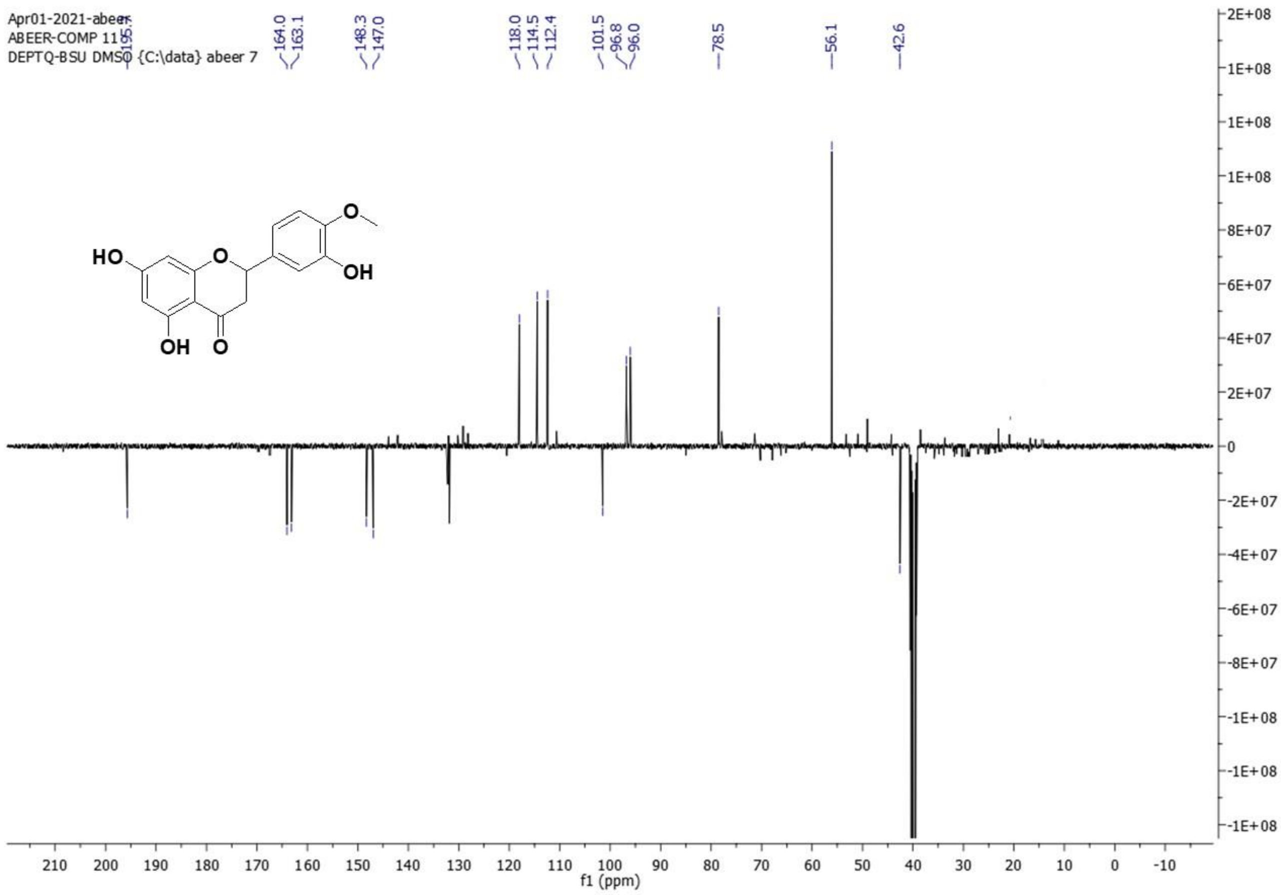

Figure S10. DEPT-Q NMR spectrum of compound **5** measured in DMSO-d_6 at 100 MHz.

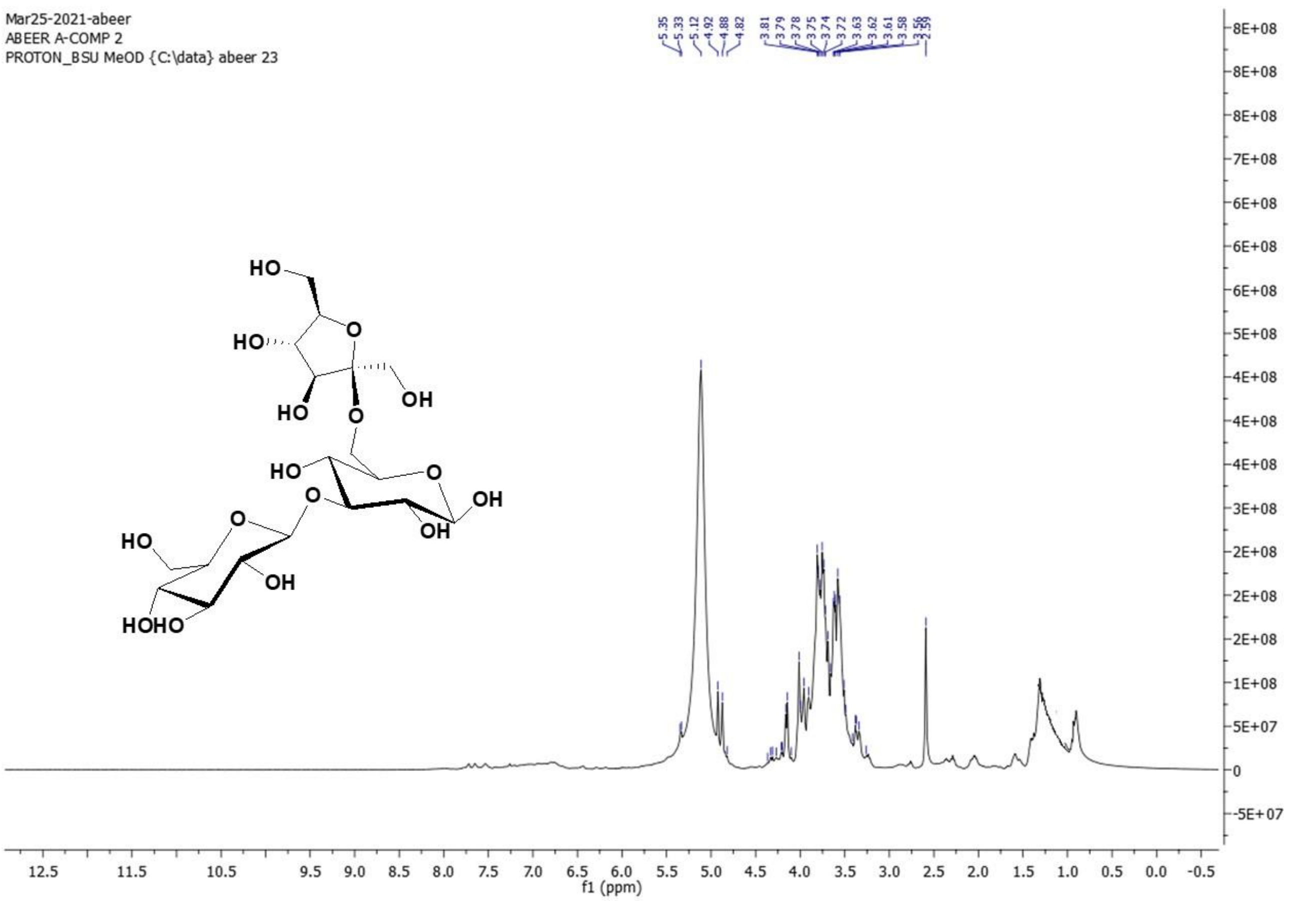

Figure S11. 1H NMR spectrum of compound **6** measured in CD_3OD-d_4 at 400 MHz.

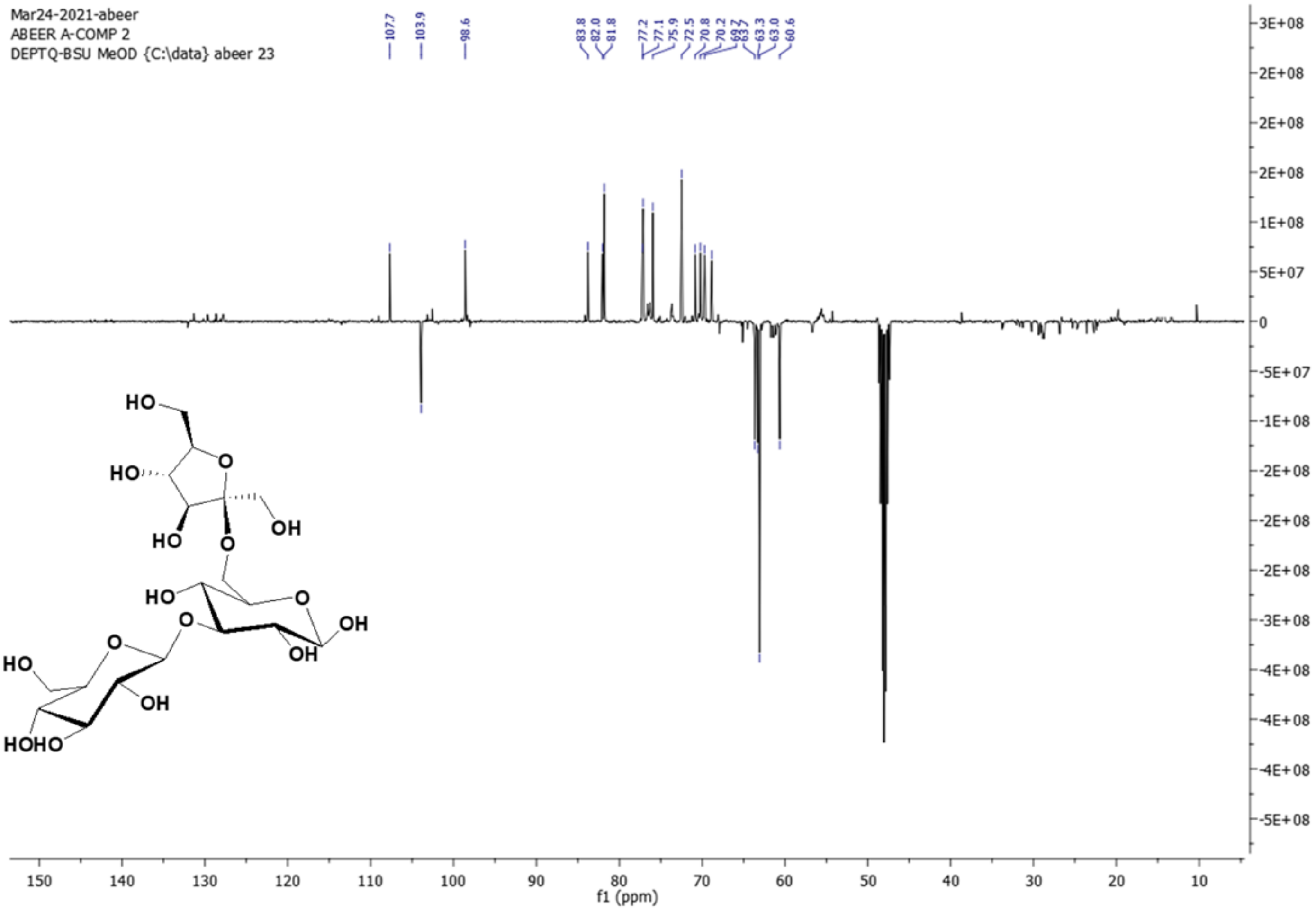

Figure S12. DEPT-Q NMR spectrum of compound **6** measured in CD_3OD-d_4 at 100 MHz.

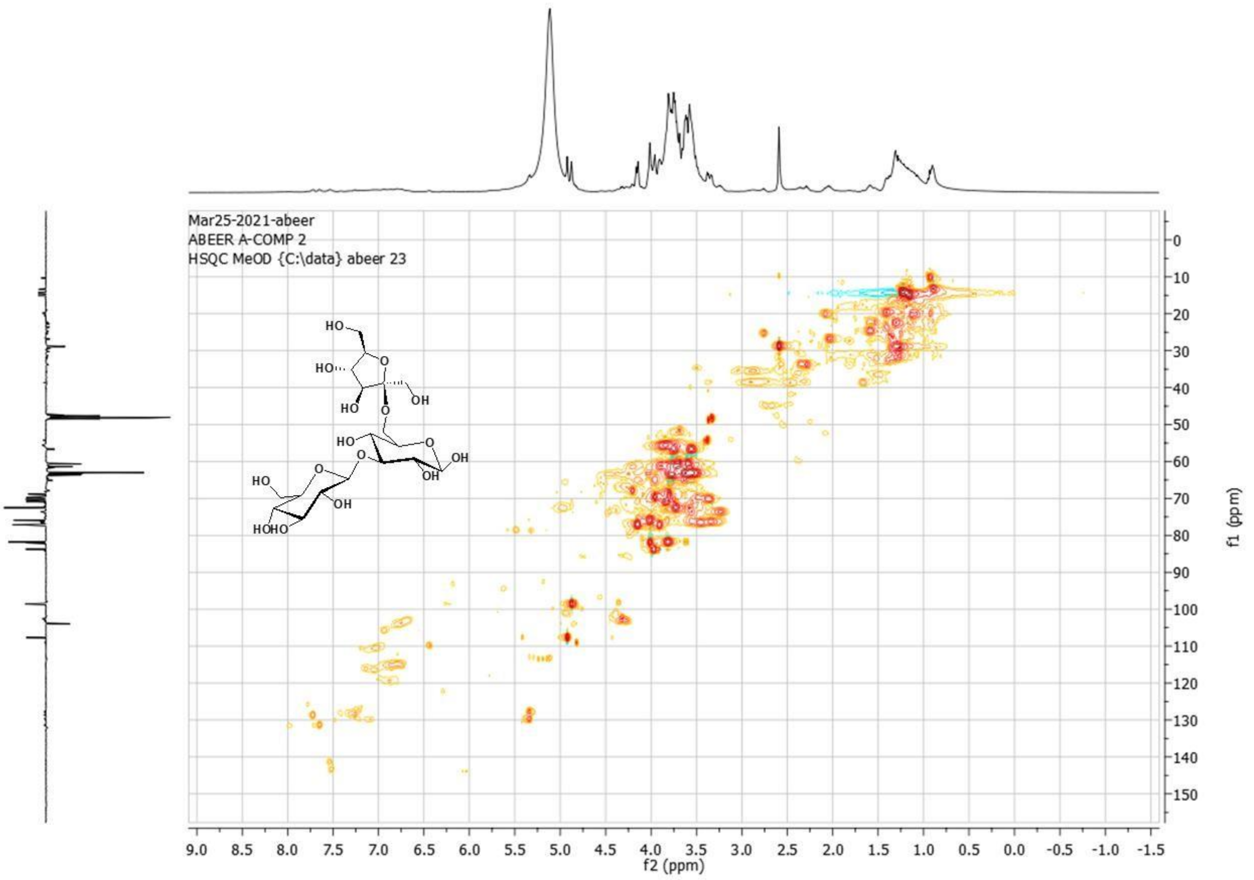

Figure S13. HSQC spectrum of compound **6** measured in CD_3OD-*d*$_4$.

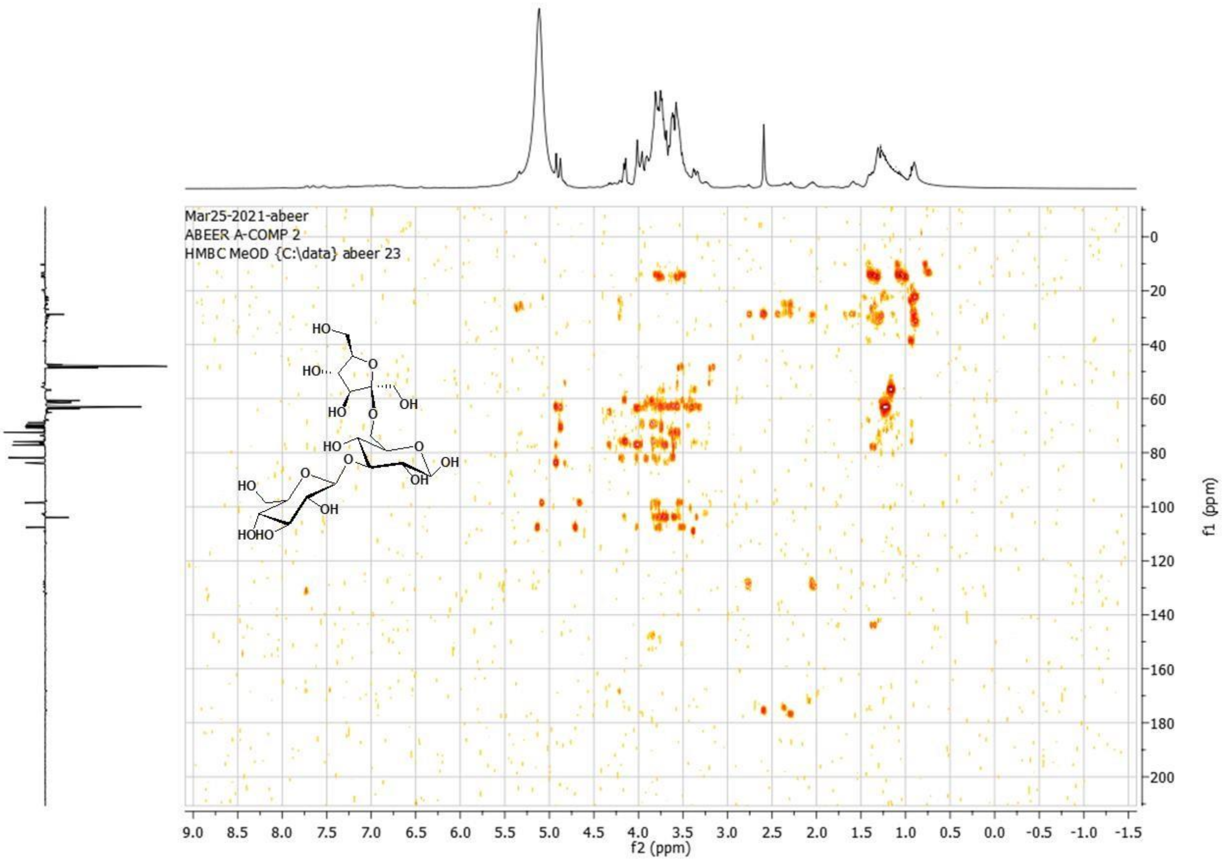

Figure S14. HMBC spectrum of compound **6** measured in CD_3OD-*d*$_4$.

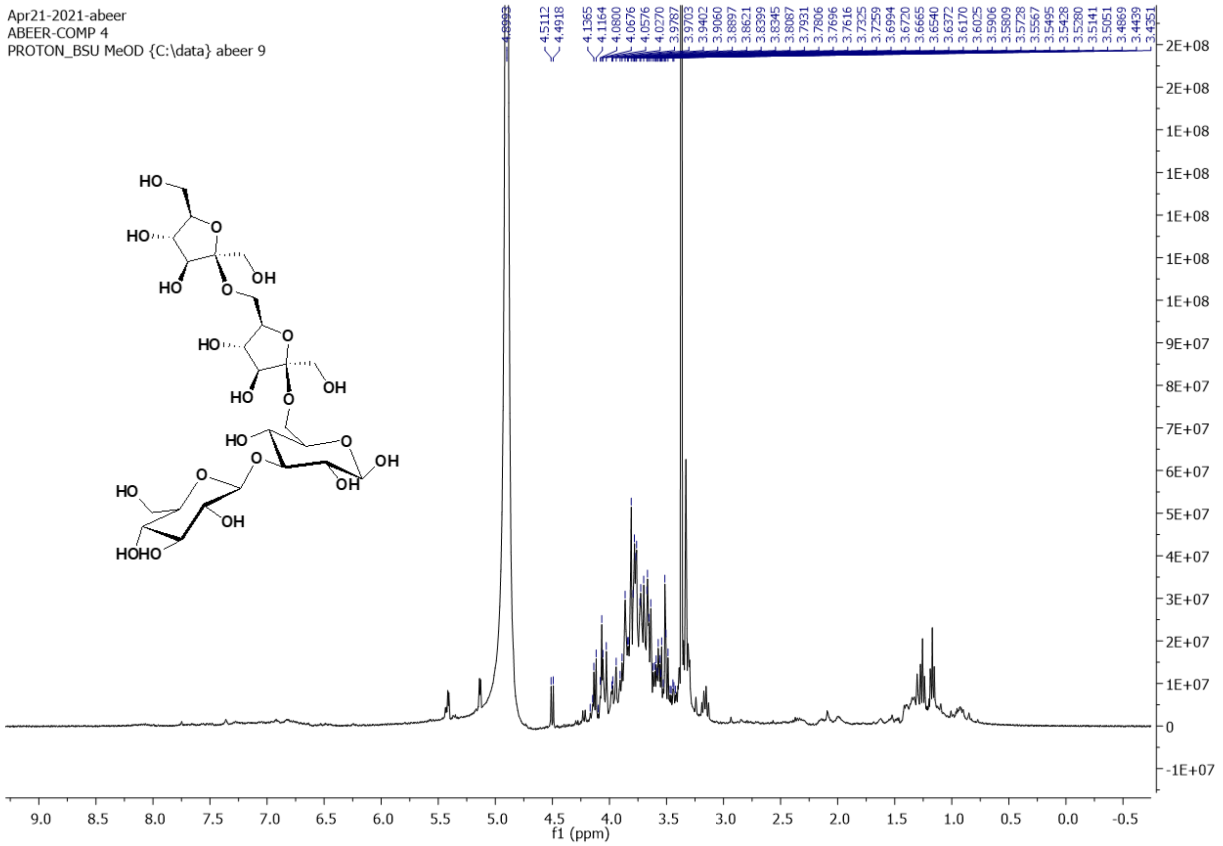

Figure S15. 1H NMR spectrum of compound **7** measured in CD_3OD-d_4 at 400 MHz.

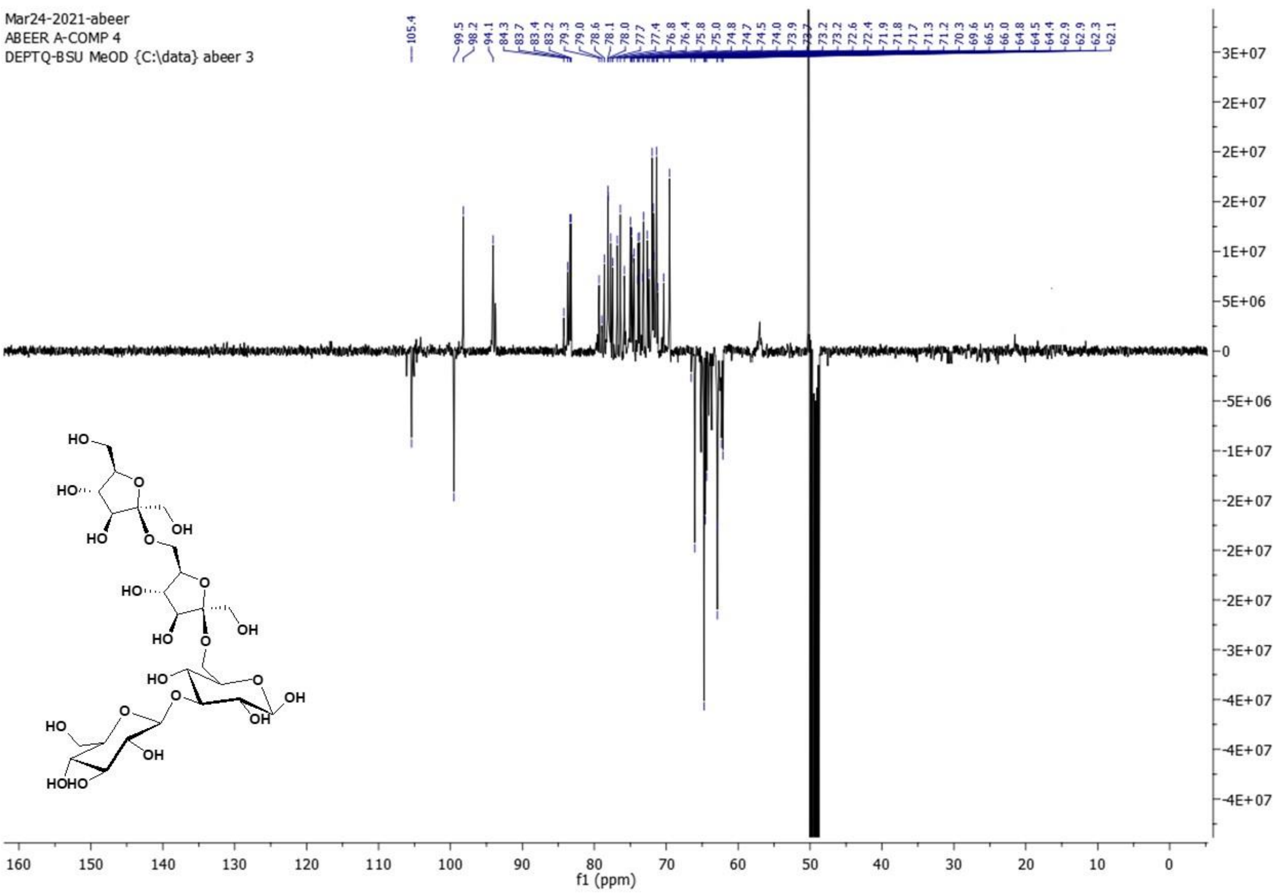

Figure S16. DEPT-Q NMR spectrum of compound **7** measured in CD_3OD-d_4 at 100 MHz.

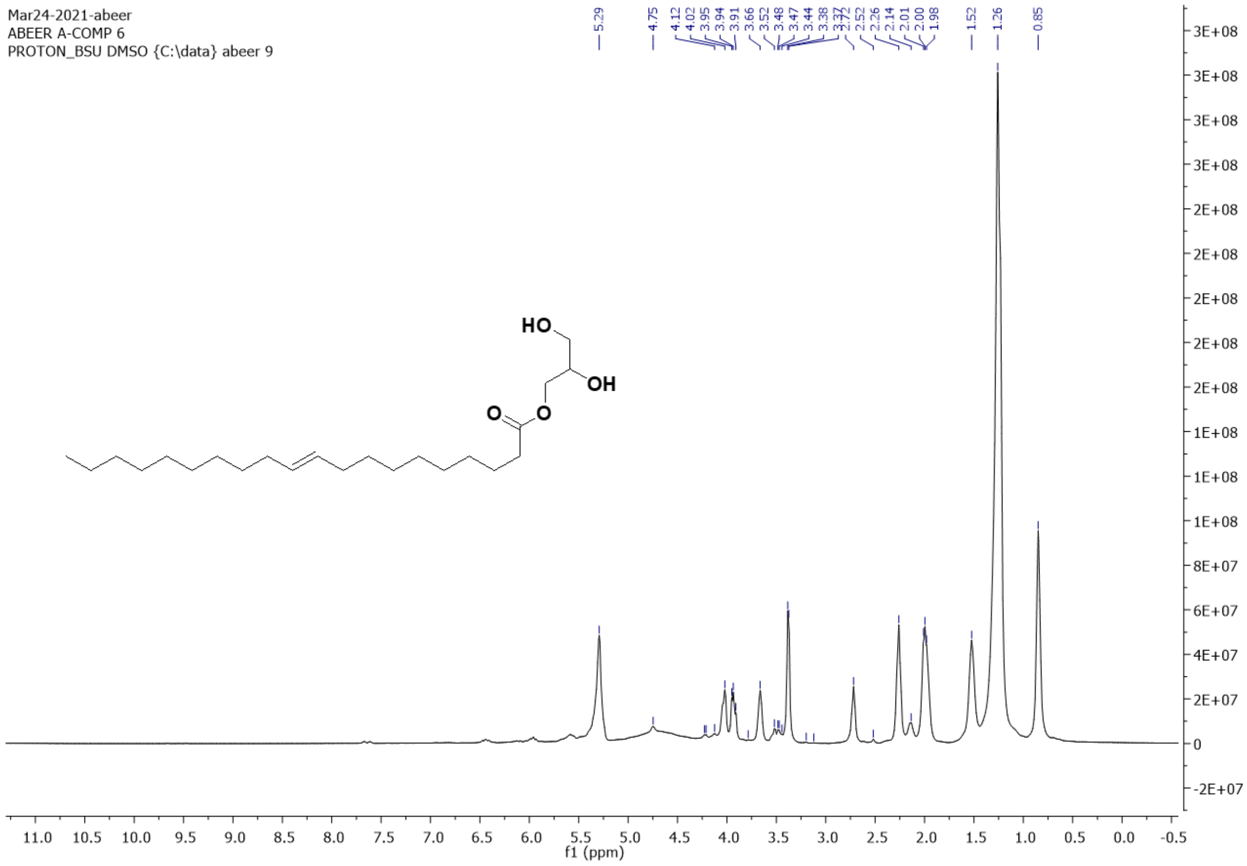

Figure S17. 1H NMR spectrum of compound **8** measured in DMSO-d_6 400 MHz.

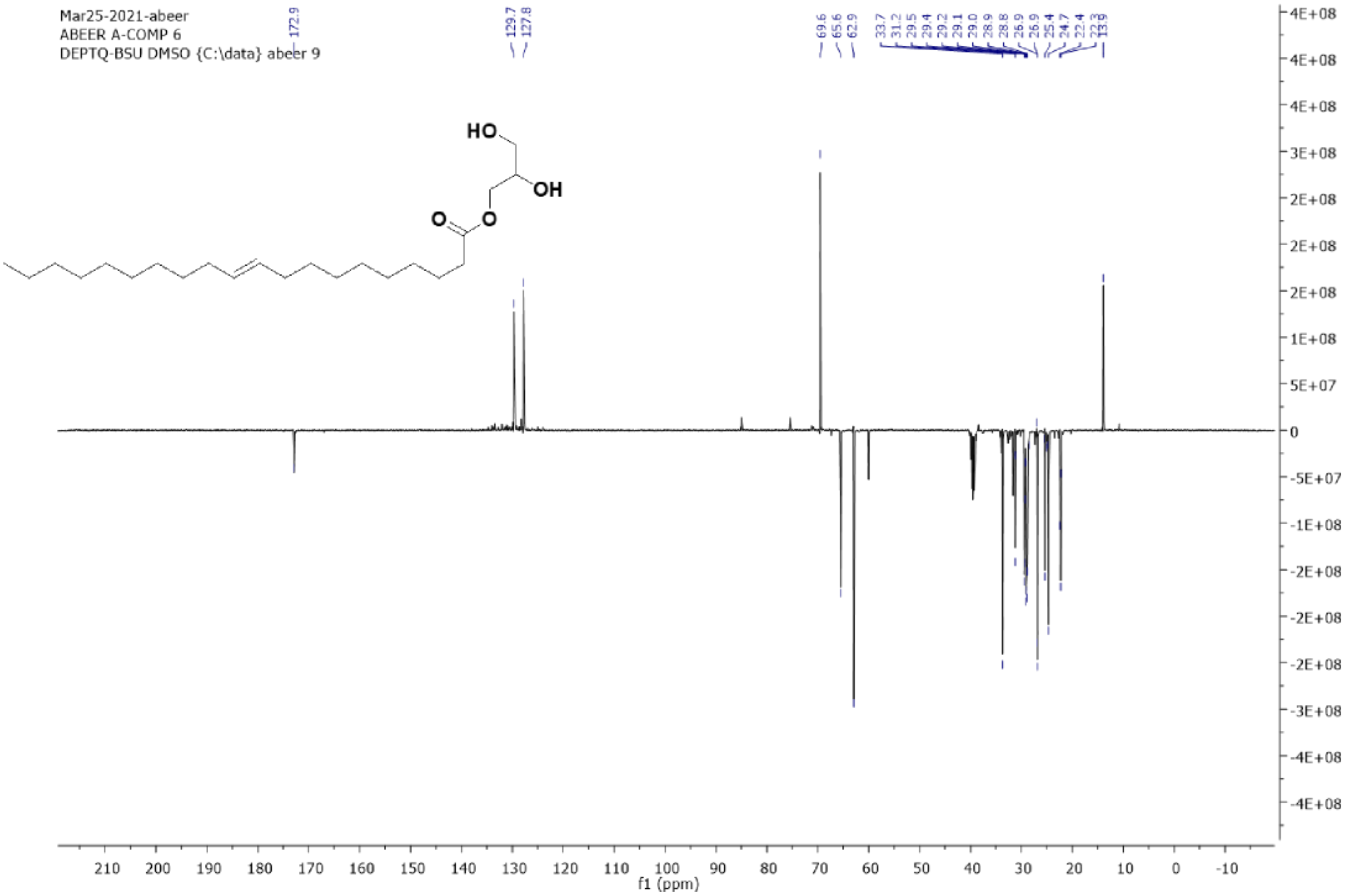

Figure S18. DEPT-Q NMR spectrum of compound **8** measured in DMSO-d_6 at 100 MHz.

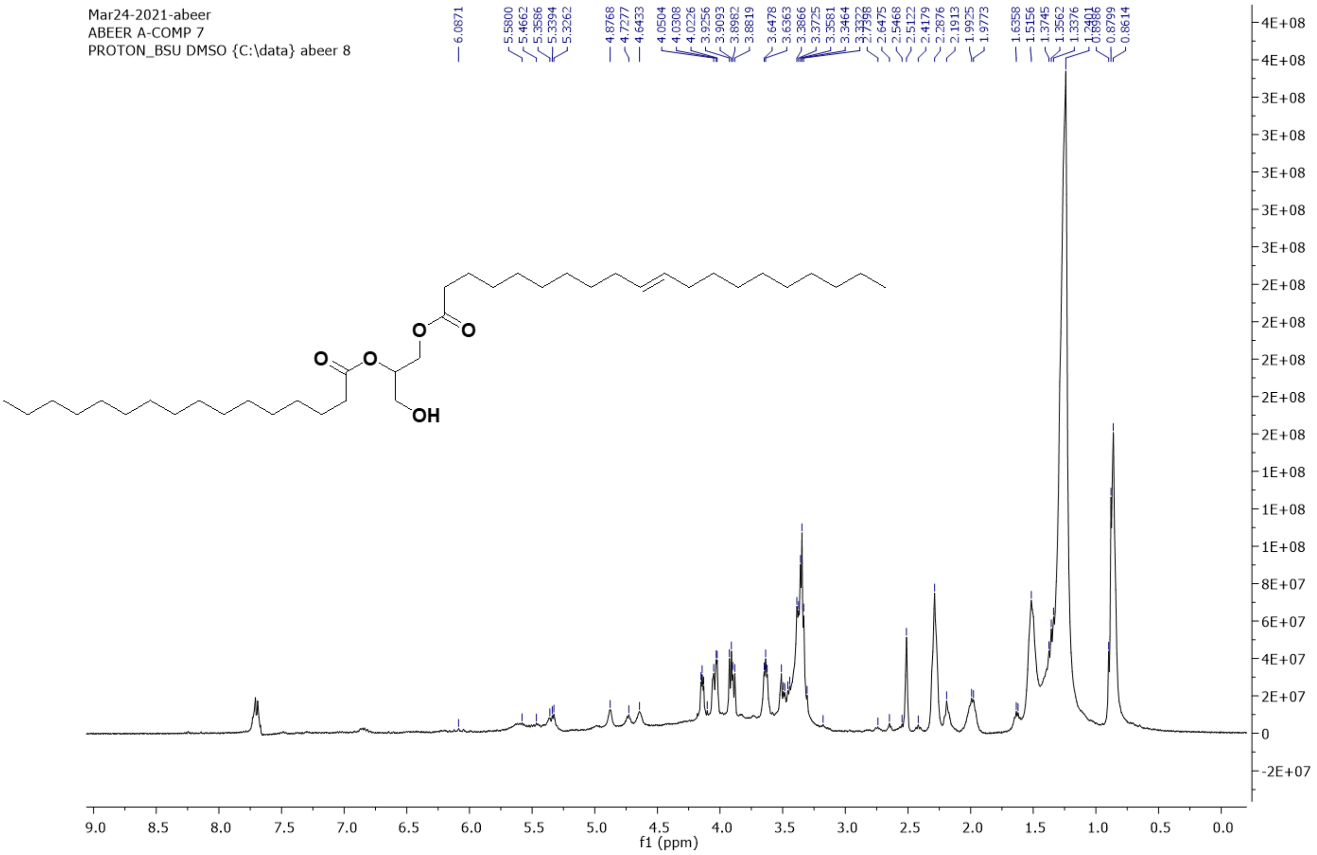

Figure S19. 1H NMR spectrum of compound **9** measured in DMSO-d_6 at 400 MHz.

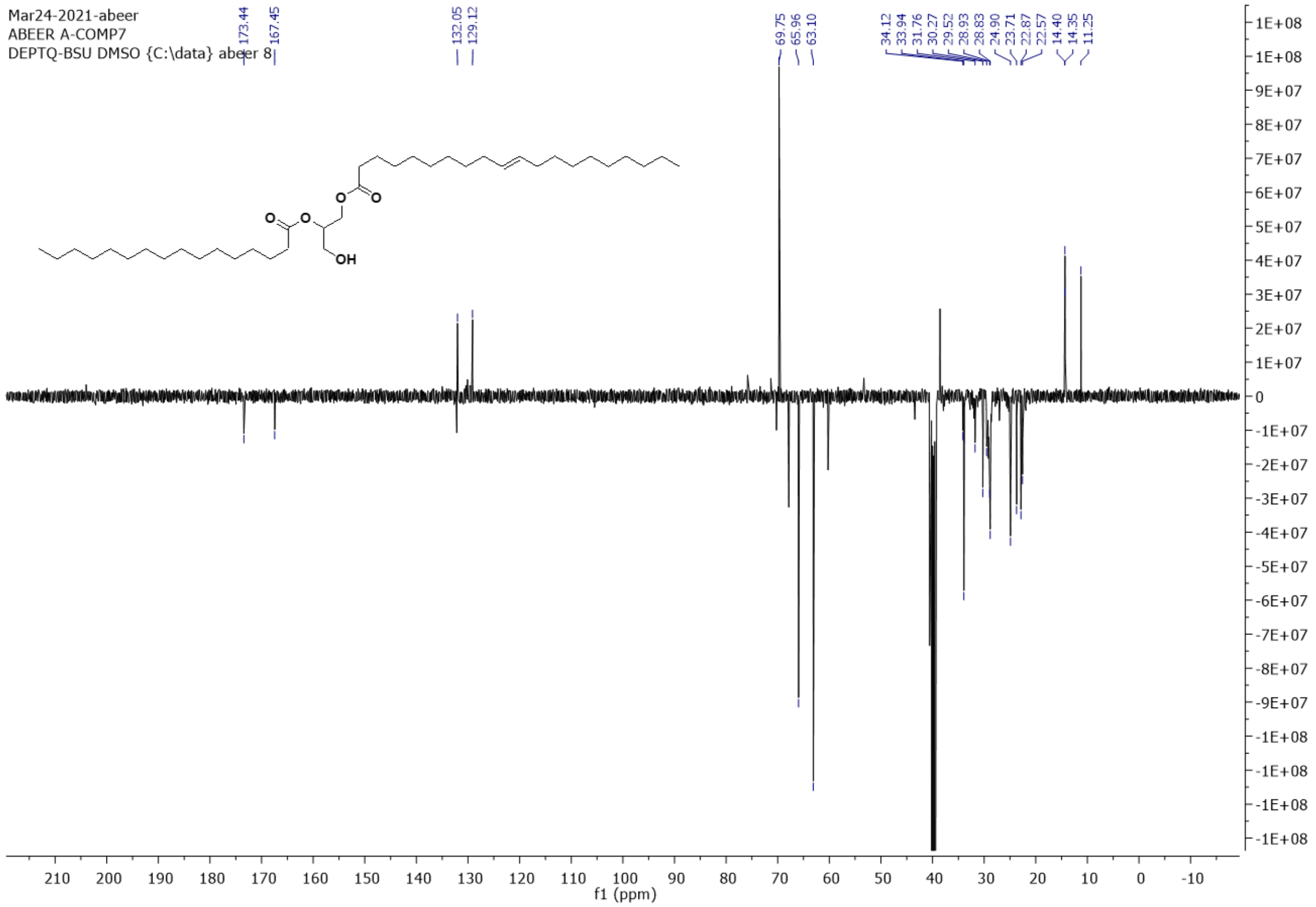

Figure S20. DEPT-Q NMR spectrum of compound **9** measured in DMSO-d_6 at 100 MHz.

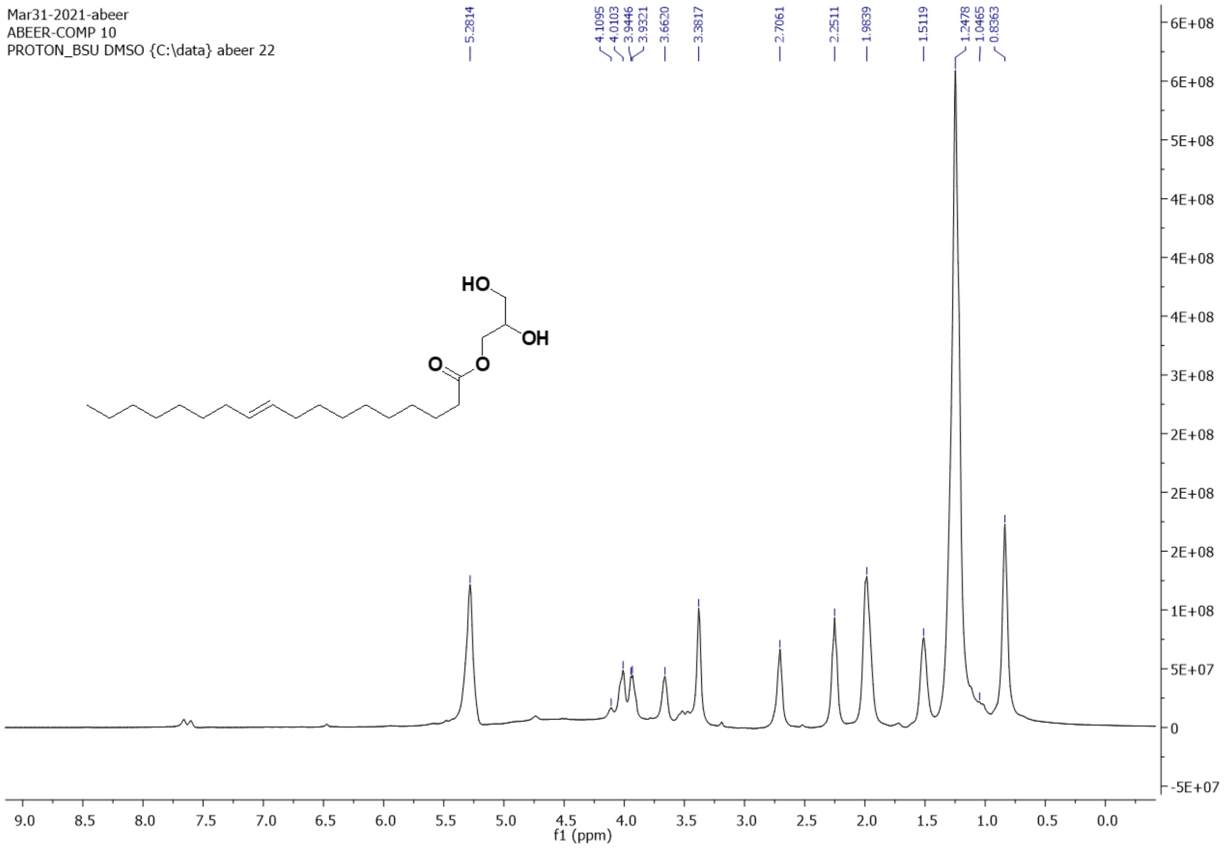

Figure S21. 1H NMR spectrum of compound **10** measured in DMSO-d_6 at 400 MHz.

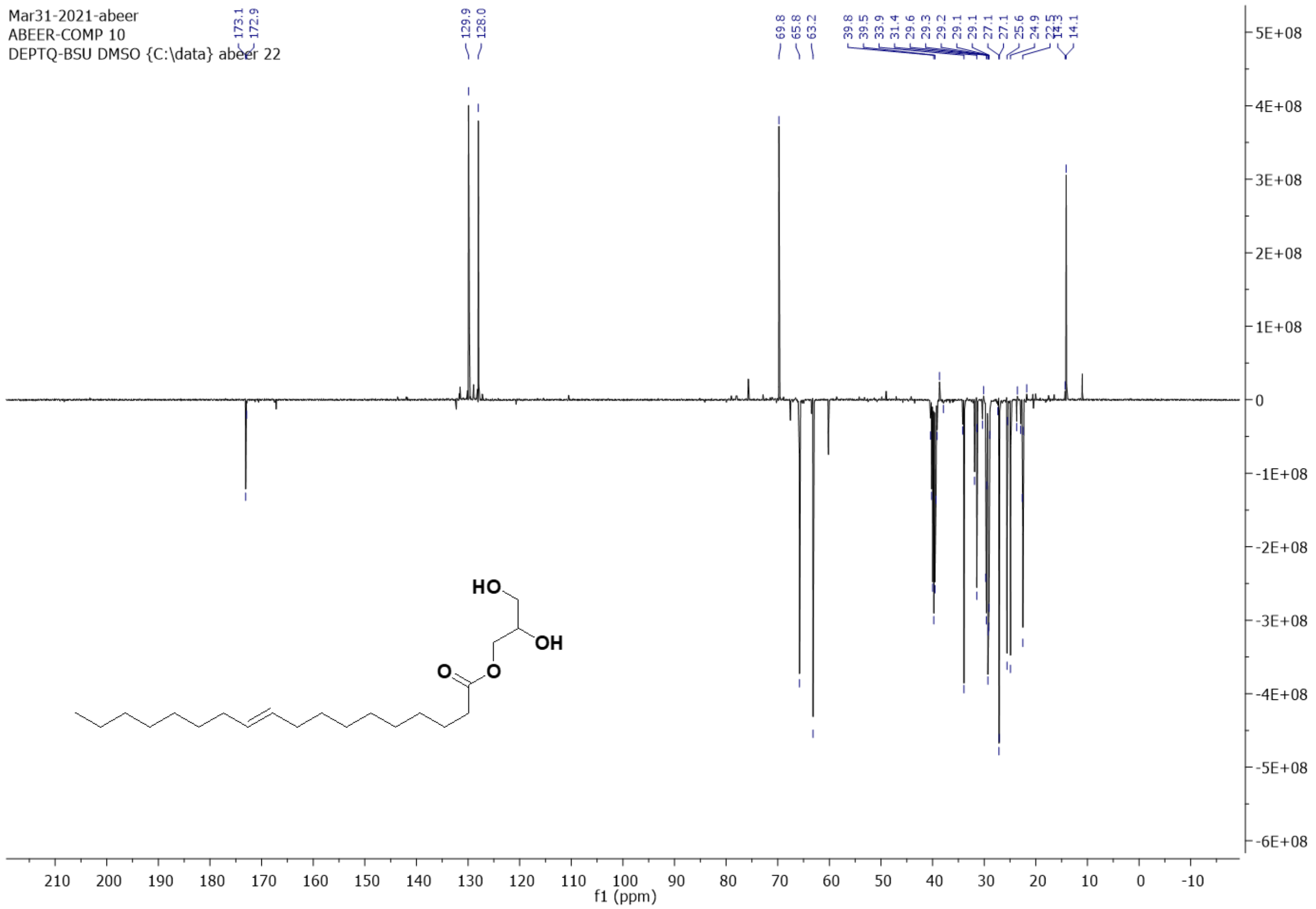

Figure S22. DEPT-Q NMR spectrum of compound **10** measured in DMSO-d_6 at 100 MHz.

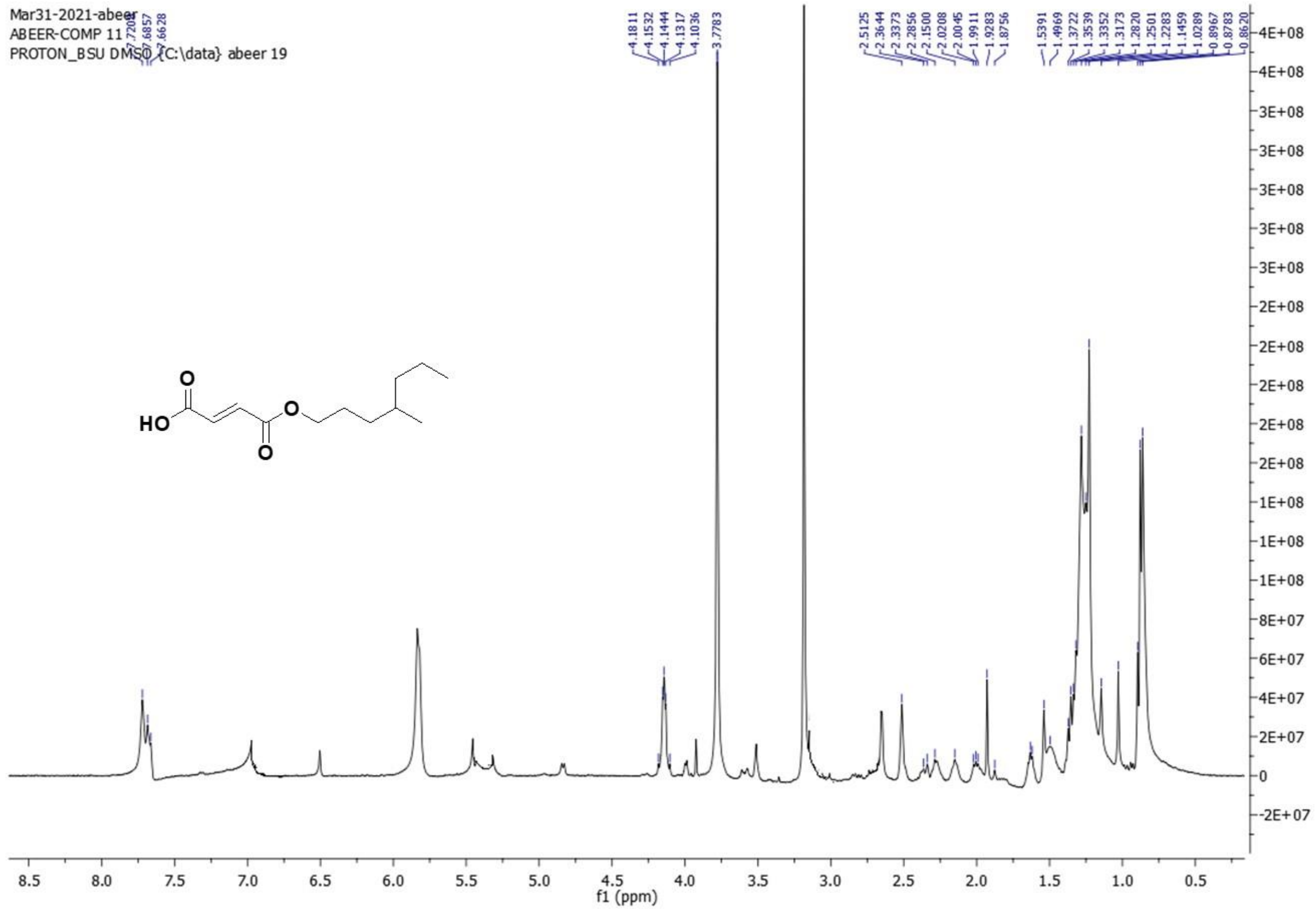

Figure S23. 1H NMR spectrum of compound **11** measured in DMSO-d_6 at 400 MHz.

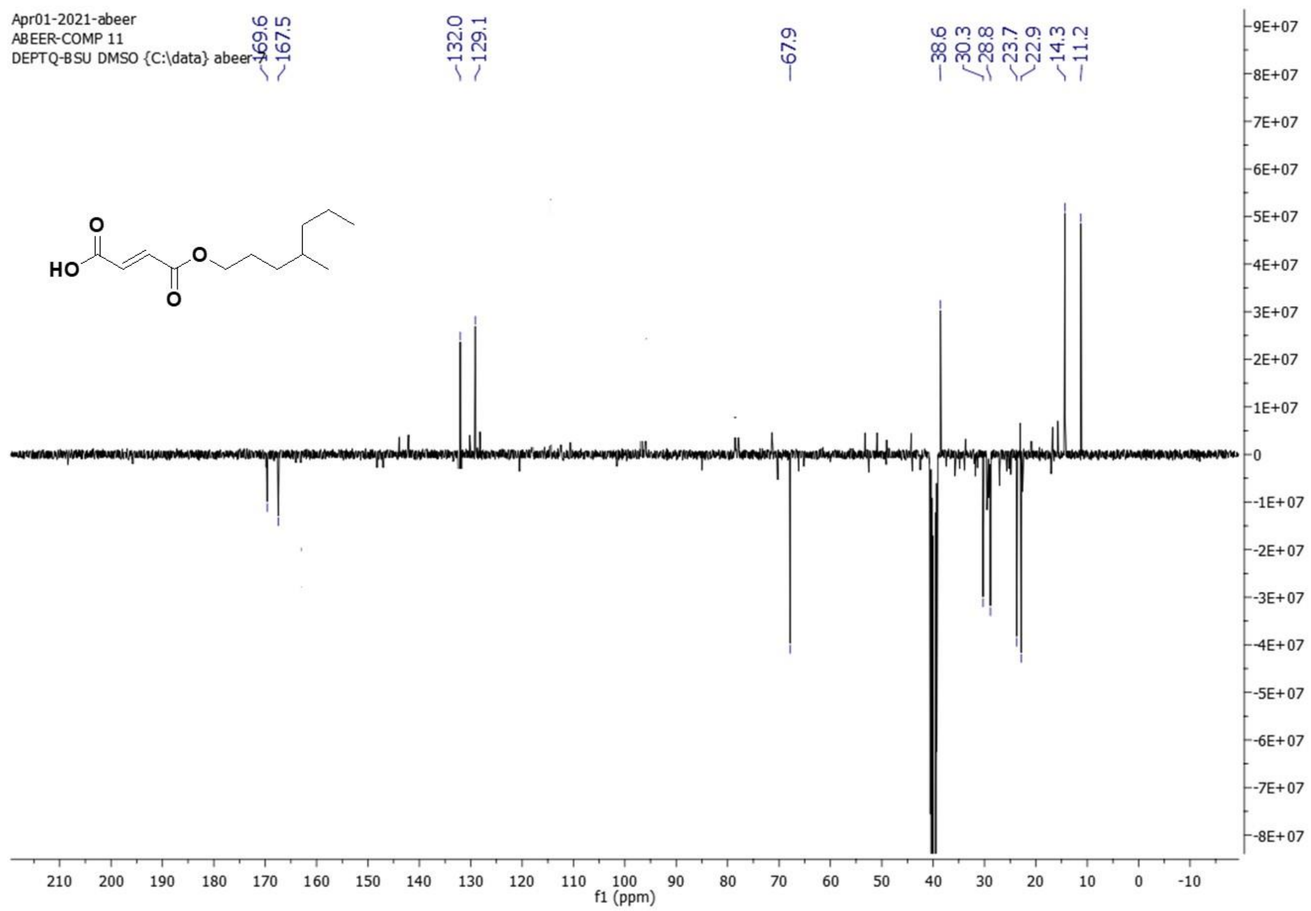

Figure S24. DEPT-Q NMR spectrum of compound **11** measured in DMSO-d_6 at 100 MHz.

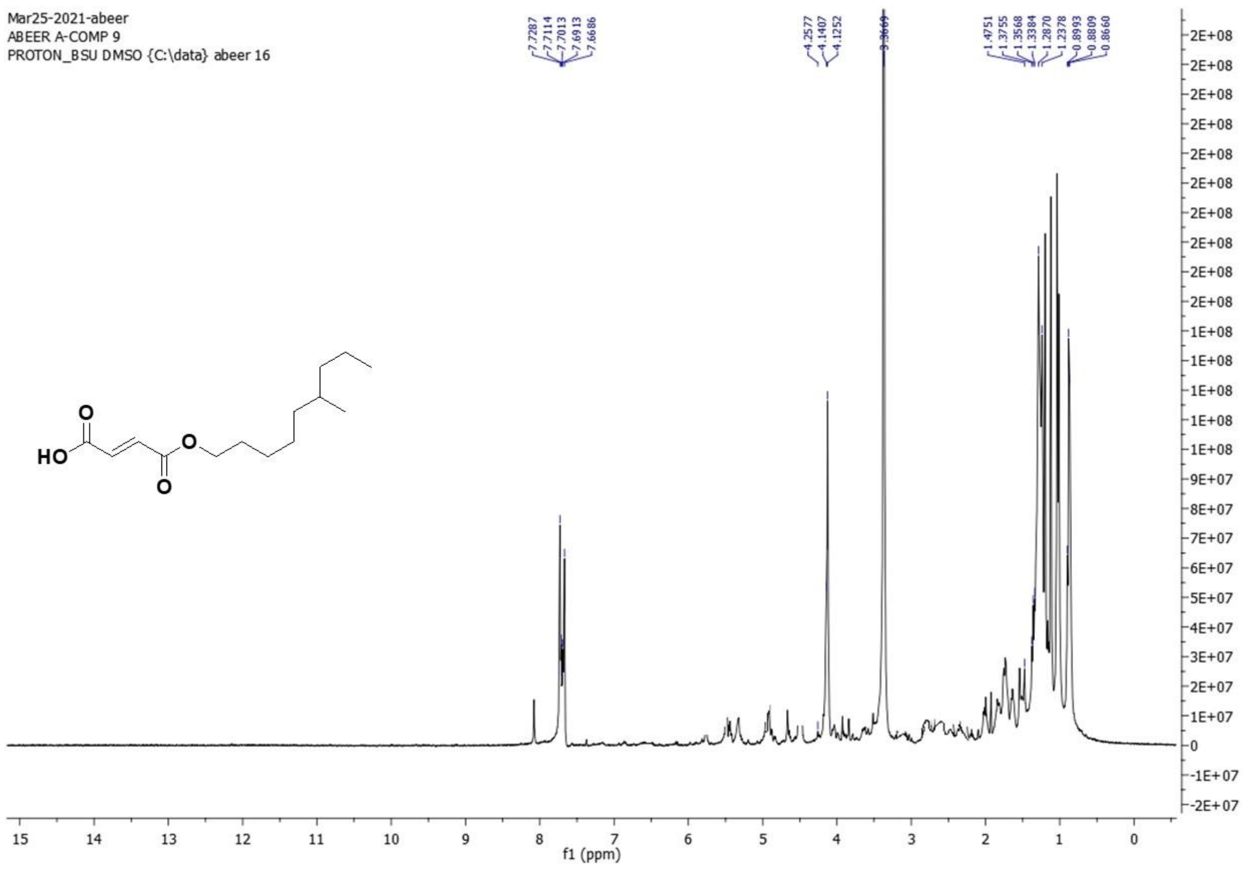

Figure S25. 1H NMR spectrum of compound **12** measured in CDCL3-*d* at 400 MHz.

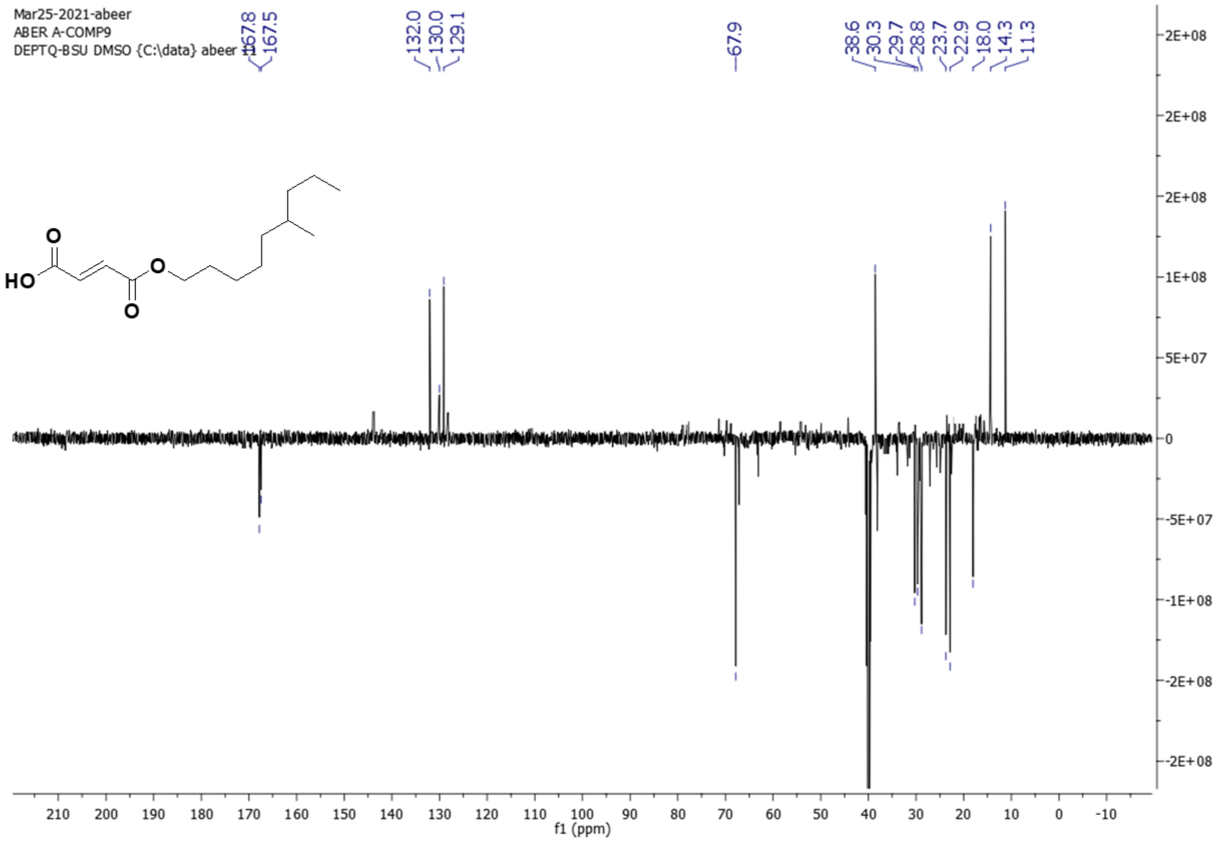

Figure S26. DEPT-Q NMR spectrum of compound **12** measured in $CDCL_3$-*d* at 100 MHz.

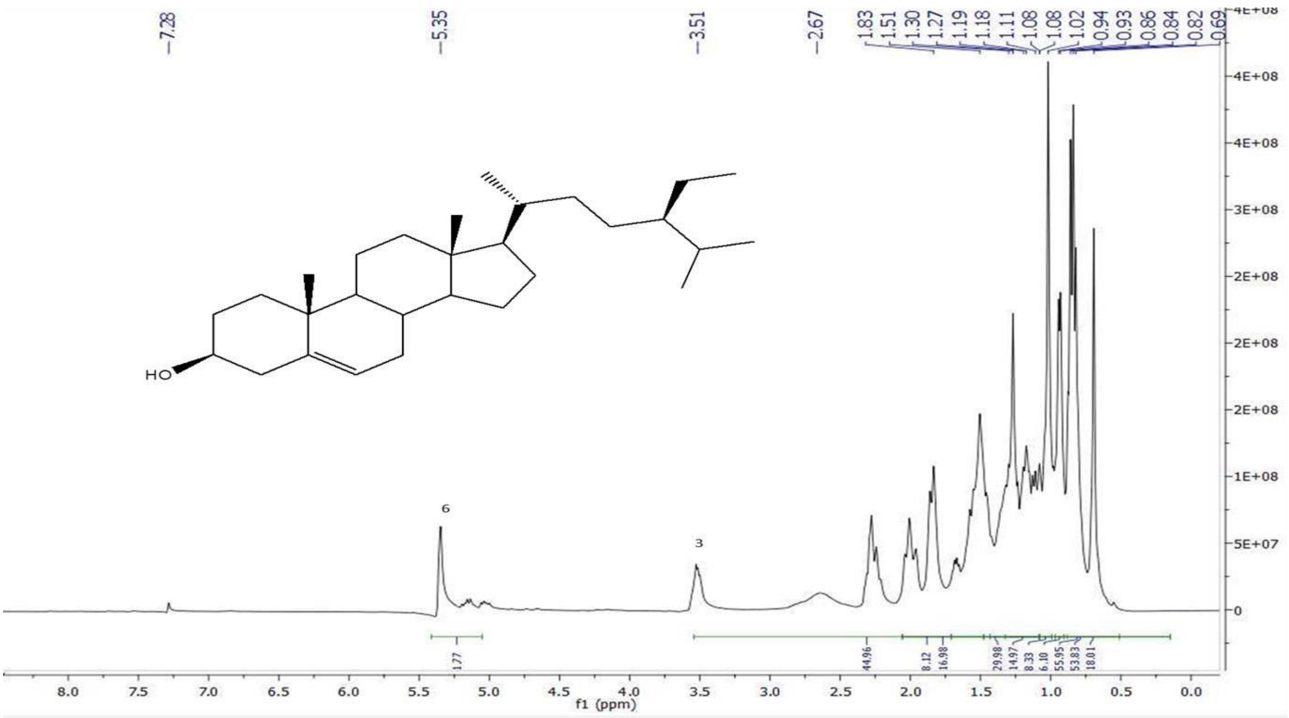

Figure S27. 1H NMR spectrum of compound **13** measured in CDCL3-*d* at 400 MHz.

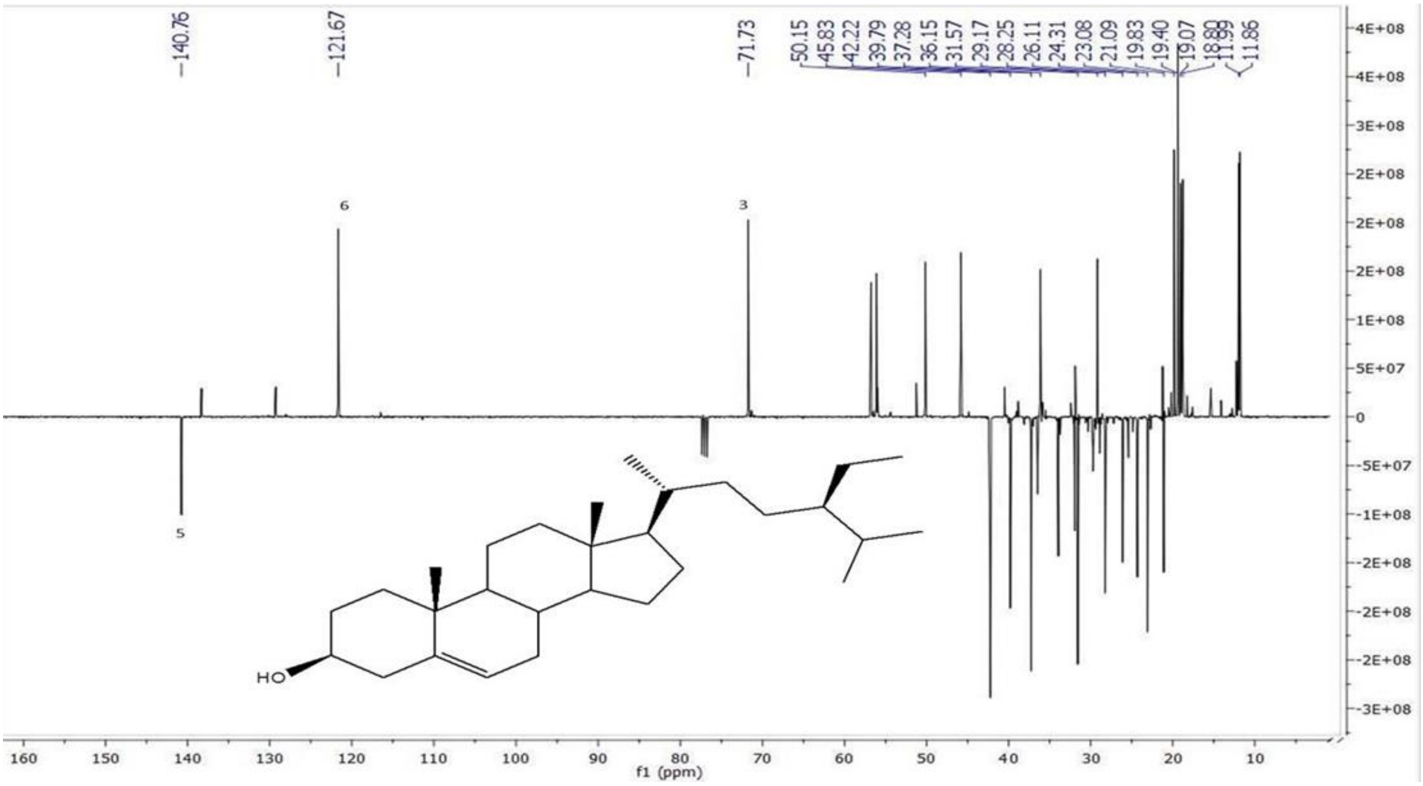

Figure S28. DEPT-Q NMR spectrum of compound **13** measured in CDCL3-*d* at 100 MHz.
